# A Conditional Dual-Branch Cross-Fusion Network with Cross-Modal Attention for Vibro-Acoustic Engine Fault Diagnosis

**DOI:** 10.3390/s26216778

**Published:** 2026-10-26

**Authors:** Hui Wang, Xiaolong Zhu, Junhong Zhang, Qihua Wu, Jiewei Lin

**Affiliations:** 1 State Key Laboratory of Engines, Tianjin University, Tianjin 300072, China; 1019201127@tju.edu.cn (H.W.); zhangjh@tju.edu.cn (J.Z.); 1123201047@tju.edu.cn (Q.W.); 2 Weichai Power Co., Ltd., Weifang 261001, China; 3 School of Information and Intelligent Engineering, Tianjin Renai College, Tianjin 300072, China; zhuxl@tju.edu.cn

**Keywords:** diesel-engine fault diagnosis, conditional attention, cross-fusion network, thermal faults, mechanical faults, vibro-acoustic fusion

## Abstract

**Highlights:**

**What are the main findings?**

- The best CDCFN variants achieved mean accuracies of 98.23 ± 0.68% for thermal faults and 97.99 ± 0.63% for mechanical faults with 50 training samples per class.
- Under matched Gaussian-noise training and testing at −12 dB, CDCFN_A/V achieved across-load mean accuracies of 94.89 ± 0.34% and 74.71 ± 1.45% on the thermal- and mechanical-fault datasets, respectively.

**What are the implications of the main findings?**

- Conditional cross-modal weighting improved performance across the tested operating and noise conditions.
- LayerCAM connected model decisions with valve seating, piston reversal, and combustion responses.

**Abstract:**

Reliable diagnosis of diesel-engine thermal and mechanical faults remains difficult when labelled samples are scarce, operating conditions vary, and measurements are strongly contaminated by noise. A conditional dual-branch cross-fusion network with cross-modal attention (CDCFN) is proposed for joint analysis of vibration and acoustic signals. Modality-specific convolutional modules extract fault-related features. A multimodal cross-attention mechanism applies temporal attention separately to the two modalities, concatenates the attended features for joint channel attention, and then separates the reweighted streams. A cross-modal conditional-decision module forms two directional decisions by using one modality to weight the other. CDCFN was evaluated on thermal- and mechanical-fault datasets using 50 training and 100 test sample pairs per class. Across the tested load conditions, the best mean accuracies were 98.23 ± 0.68% for CDCFN_V/A on the thermal-fault dataset and 97.99 ± 0.63% for CDCFN_A/V on the mechanical-fault dataset; the corresponding weighted F1 scores were 98.22% and 98.05%. Under matched Gaussian-noise training and testing at −12 dB, CDCFN_A/V achieved across-load mean accuracies of 94.89 ± 0.34% and 74.71 ± 1.45%, respectively. LayerCAM indicated attention to intake-valve seating, exhaust-valve seating, piston reversal, and combustion responses. These results support directional cross-modal attention for the tested small-sample, variable-load, and matched-noise settings.

## 1. Introduction

Diesel engines are widely used in automotive, marine, and construction applications [1]. Their components operate under coupled fluid, gas, and solid interactions, high temperature, high pressure, and strong background noise. Prolonged operation under these conditions can initiate faults that damage critical components and compromise the entire system [2,3,4]. Early and reliable fault identification is therefore important for safe and stable engine operation.

Advances in artificial intelligence and sensing have increased the use of data-driven models for machinery fault diagnosis [5,6]. These models broadly comprise conventional machine-learning methods and deep-learning methods. Machine-learning pipelines usually separate feature extraction from fault classification and can be effective for relatively simple tasks [7]. Deep-learning models jointly optimise convolutional feature extraction and fully connected classification through backpropagation, allowing the representation to adapt to the diagnostic task [8].

Conventional machine-learning studies have combined engineered signal features with shallow classifiers. Imtiaz et al. [9] used acoustic cepstral features and a support vector machine to identify 27 gasoline-engine faults under one operating condition. Kuai et al. [10] combined wavelet-packet decomposition, autospectral analysis, and a shallow neural network to detect valve-clearance faults at 1500 RPM under noise. Zhang et al. [11] used refined composite multiscale fuzzy entropy and a firefly-optimised support vector machine to diagnose valve-train faults at 1200 RPM and 50% load. For limited labelled data, Tang et al. [12] combined adaptive recursive variational mode decomposition with component-energy distribution spectra to identify valve-train and fuel-system faults at 2300 RPM and full load. Ke et al. [13] introduced multiscale bidirectional diversity entropy for diesel-injector fault diagnosis. These approaches can perform well, but their accuracy depends strongly on hand-crafted features, signal preprocessing, and predefined parameters. This dependence limits their adaptability to complex equipment and operating environments.

Deep-learning models reduce the need for separate feature engineering and have therefore attracted substantial interest. Tabaszewski et al. [14] used a convolutional neural network (CNN) to identify abnormal intake- and exhaust-valve clearances from three-axis cylinder-head vibration data collected at 700–1700 RPM. Qin et al. [15] combined multiscale convolution, long short-term memory (LSTM) units, and residual denoising for misfire diagnosis at 1300, 1800, and 2200 RPM. The model was more robust to noise and operating-condition changes than DNN, CNN, and multiscale CNN baselines. Nguyen Thanh and Cho [16] developed a CNN–bidirectional LSTM model for anomaly detection in 125 and 250 kW industrial diesel generators. To improve physical interpretability, Singh et al. [17] embedded engine models within a one-dimensional CNN and evaluated the framework at 1000–1600 RPM. Li et al. [18] introduced prior-distribution-guided sparse attention for cross-domain feature mining in the time, frequency, and envelope domains. Chu et al. [19] used hybrid attention to fuse multi-cylinder vibration features and reported mean accuracies of 100% for known faults and 93.78% for single-cylinder fault recognition.

These studies show that well-designed deep networks can perform strongly when sufficient representative samples are available. In industrial applications, however, labelled fault data are often scarce. Cui et al. [20] addressed limited samples by combining generative adversarial data augmentation with transfer learning for single and compound fuel-system faults. Zhang et al. [21] proposed a wide-kernel CNN-LSTM model to improve robustness across fault types, equipment types, severities, and operating conditions. Zhong et al. [22] used a deep concentric Siamese network and metric learning to improve the separation of difficult samples in small-sample diagnosis. Although these strategies reduce data requirements, performance can still deteriorate when operating conditions change and response distributions shift.

Diesel-engine responses vary nonlinearly with speed and load because fuel injection and combustion change across operating conditions. Guo et al. [23] proposed a two-stage domain-adaptation method with depthwise separable convolution and achieved mean accuracy above 90% for fuel-, valve-train-, and cooling-system faults across four load conditions. Sun et al. [24] combined convolution with multi-head-attention LSTM units to extract local and global features under navigation, manoeuvring, and anchoring conditions. Hu et al. [25] developed an acoustic-emission model for cross-condition and cross-engine diagnosis of exhaust-valve leakage. These studies improve generalisation, but most analyse a single sensing modality or require explicit domain adaptation.

Signals acquired from different engine locations and sensing modalities contain correlated but non-identical information. Multimodal fusion can therefore provide a more complete representation of engine condition. Li et al. [26] proposed a CNN-based multimodal-input Transformer for misfire diagnosis and reported 99.536% mean accuracy at an SNR of −4 dB. Liu et al. [27] fused multi-sensor information using an adaptive dynamic weighted hybrid-distance Taguchi method. Qin et al. [28] combined time- and frequency-domain vibration features in a dual convolutional network for misfire diagnosis under four operating conditions. Zhao et al. [29] segmented signals according to the intake, compression, combustion, and exhaust processes and combined multi-branch CNN outputs by ensemble learning. Vibro-acoustic fusion is physically motivated because structural vibration and radiated sound share excitation sources but follow different transmission paths [2]. Zhu et al. [30] exploited this relationship in MJA-Net, which used joint attention to fuse vibration and acoustic features across four load conditions. Their results showed that multimodal fusion outperformed either modality alone.

Recent studies further illustrate complementary roles for attention-based diagnosis and neural modelling. Wu et al. [31] combined time–frequency convolution with sparse attention for secondary-suspension diagnosis; their method operates on time–frequency representations, whereas CDCFN investigates reciprocal weighting between synchronised vibration and acoustic modalities. Molaie et al. [32] used neural networks to estimate ISO-based gear safety factors, representing mechanical-condition prediction rather than sensor-based fault classification. Mo et al. [33] addressed limited fault samples and changing operating conditions through multi-cylinder similarity and memory augmentation; CDCFN instead focuses on paired vibro-acoustic fusion.

Existing methods nevertheless face three related limitations: First, strong noise can reduce diagnostic accuracy across operating conditions. Second, deep models often require more labelled fault data than are available in practice. Third, vibration and acoustic measurements contain fault-dependent correlations that are not fully exploited by conventional fusion classifiers. These limitations are particularly important for small-sample diagnosis under multiple operating conditions.

To address these limitations, we propose a conditional dual-branch cross-fusion network with cross-modal attention (CDCFN) for small-sample diagnosis of intake-system and timing-gear faults under strong noise. The main contributions are as follows:

(1) CDCFN integrates attention-based feature extraction, cross-modal interaction, and conditional classification for vibration and acoustic measurements. The network is designed to recognise representative thermal and mechanical engine faults from limited samples under noisy conditions.
(2) Modality-specific convolutional modules extract vibration and acoustic features. MCAM first applies temporal attention separately to the two modalities, concatenates the attended features for joint channel attention, and then separates the reweighted streams. CMCD implements two reciprocal cross-modal attention-score pooling directions, allowing acoustic-weighted (A/V) and vibration-weighted (V/A) decisions without interpreting the attention scores as a probabilistic conditional distribution.
(3) Ablation and benchmark experiments use thermal- and mechanical-fault datasets collected at four loads. Accuracy, precision, recall, F1 score, and Cohen’s kappa quantify performance, stability, and resistance to matched additive noise across the tested conditions. The experiments also compare the two directions of cross-modal weighting.
(4) (LayerCAM visualisations are interpreted against the engine operating cycle. This analysis identifies attention to intake-valve seating, exhaust-valve seating, piston reversal, and combustion responses, providing a physical basis for interpreting the model decisions.

The remainder of this paper is organised as follows: Section 2 describes CDCFN and its components. Section 3 presents the test rig, datasets, and correspondence between vibro-acoustic responses and the engine cycle. Section 4 reports ablation, noise-resistance, benchmark, and interpretability analyses. Section 5 summarises the findings and limitations.

## 2. Conditional Dual-Branch Cross-Fusion Network with Cross-Modal Attention

### 2.1. Multi-Head Attention

**Figure 1:**
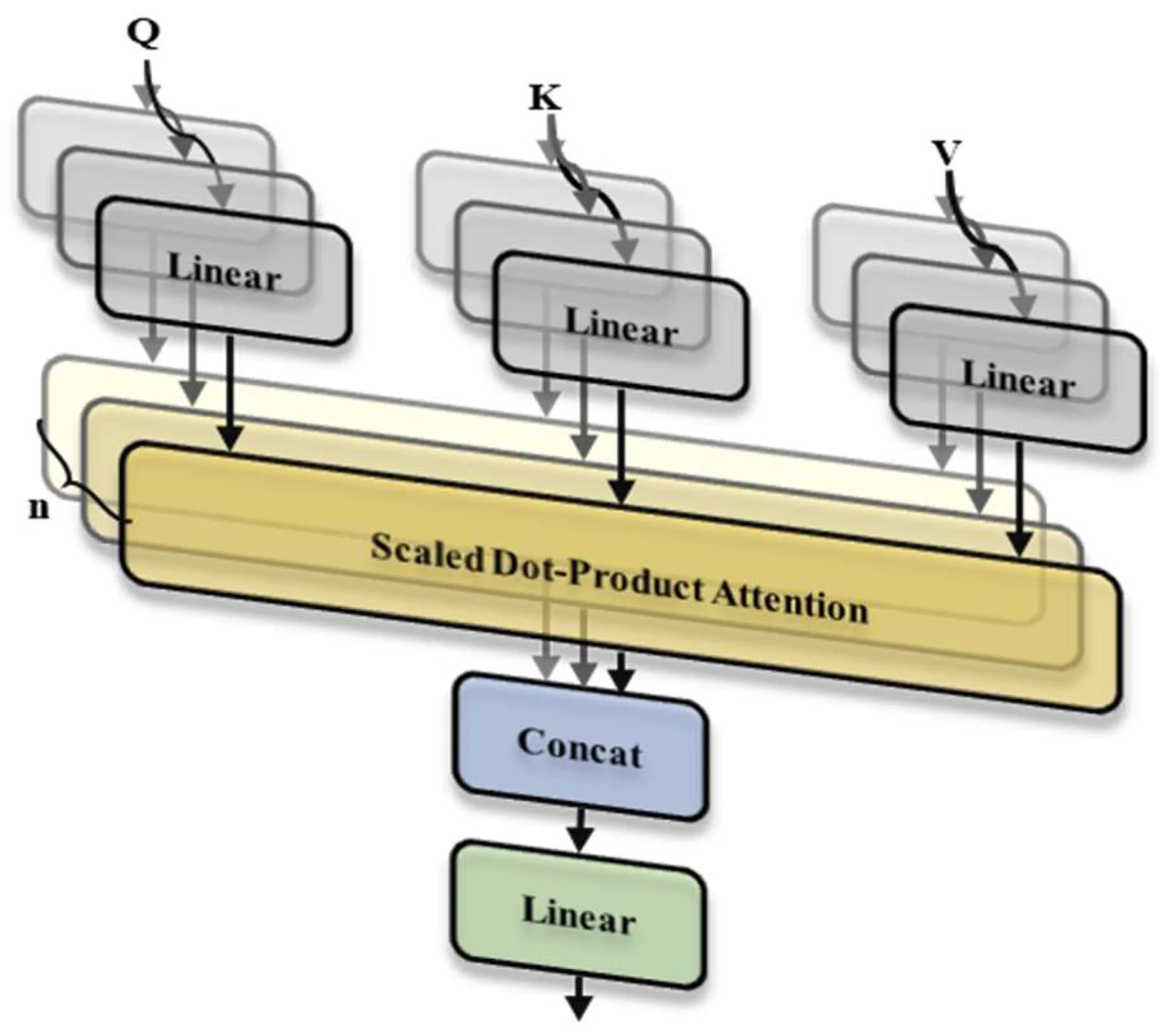
Multi-head attention mechanism.

Self-attention models dependencies among elements of the same input and reduces reliance on external information [34]. Query (Q), key (K), and value (V) representations are obtained from different linear projections of an input X. Their interactions assign higher weights to informative elements and lower weights to less relevant elements. Multi-head attention extends this operation by learning several attention maps in parallel and concatenating their outputs, as shown in Figure 1. This structure reduces the tendency of a single attention head to concentrate on only one subset of features.

The input sequence was projected linearly to Q, K, and V using three weight matrices, $W^{Q},W^{K},W^{V}$. Each projection was partitioned into multiple heads, and scaled dot-product attention was computed independently for each head [35]:

(1) $$Attention(Q,K,V)=Softmax\left(\frac{Q\cdot K^{T}}{\sqrt{d_{k}}}\right)\cdot V$$

The outputs from all attention heads were concatenated and passed through a linear layer. This parallel representation captures complementary dependencies from several subspaces. In simpler configurations, Q=K=V, so the same features serve as Q, K, and V. The choice of projections was matched to the diagnostic architecture.

### 2.2. CDCFN Framework

**Figure 2:**
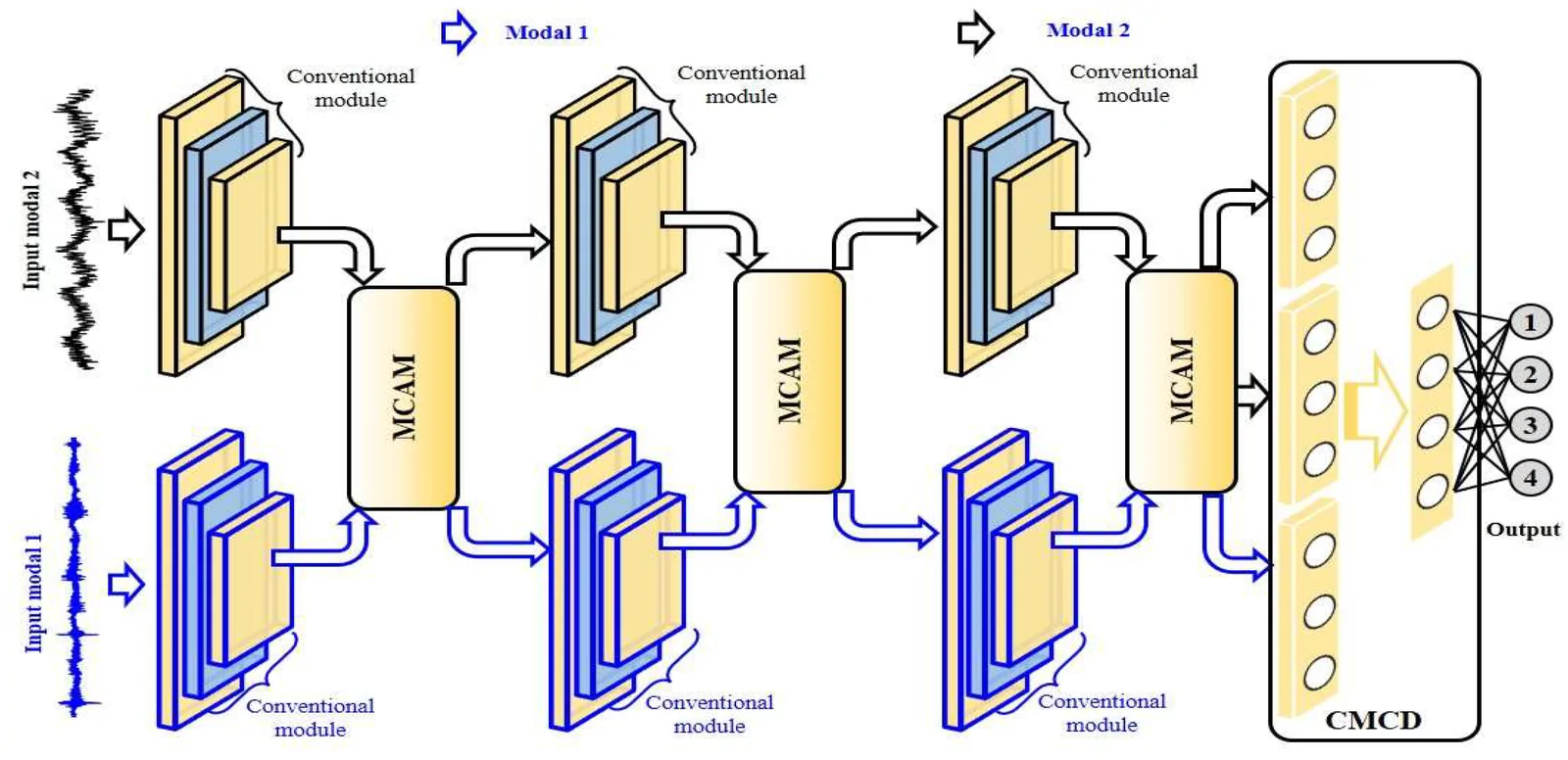
Architecture of CDCFN.

Acoustic and vibration signals provide complementary measurements of engine operation. Near-field sound and surface vibration share excitation sources, but their transmission paths differ [2]. CDCFN is designed to extract fault-related information and promote interaction between these modalities across the tested operating conditions. As shown in Figure 2, the network contains three feature-extraction stages and a classification stage. Each extraction stage comprises separate convolutional modules for vibration and acoustic inputs, followed by MCAM. Within MCAM, temporal attention is applied separately to each modality. The two attended feature maps are then concatenated along the channel dimension, jointly reweighted by channel attention, and separated again into vibration and acoustic streams. Finally, CMCD forms a directional decision vector through cross-modal attention-score pooling in either the A/V or V/A direction.

#### 2.2.1. Multimodal Cross-Attention Mechanism

**Figure 3:**
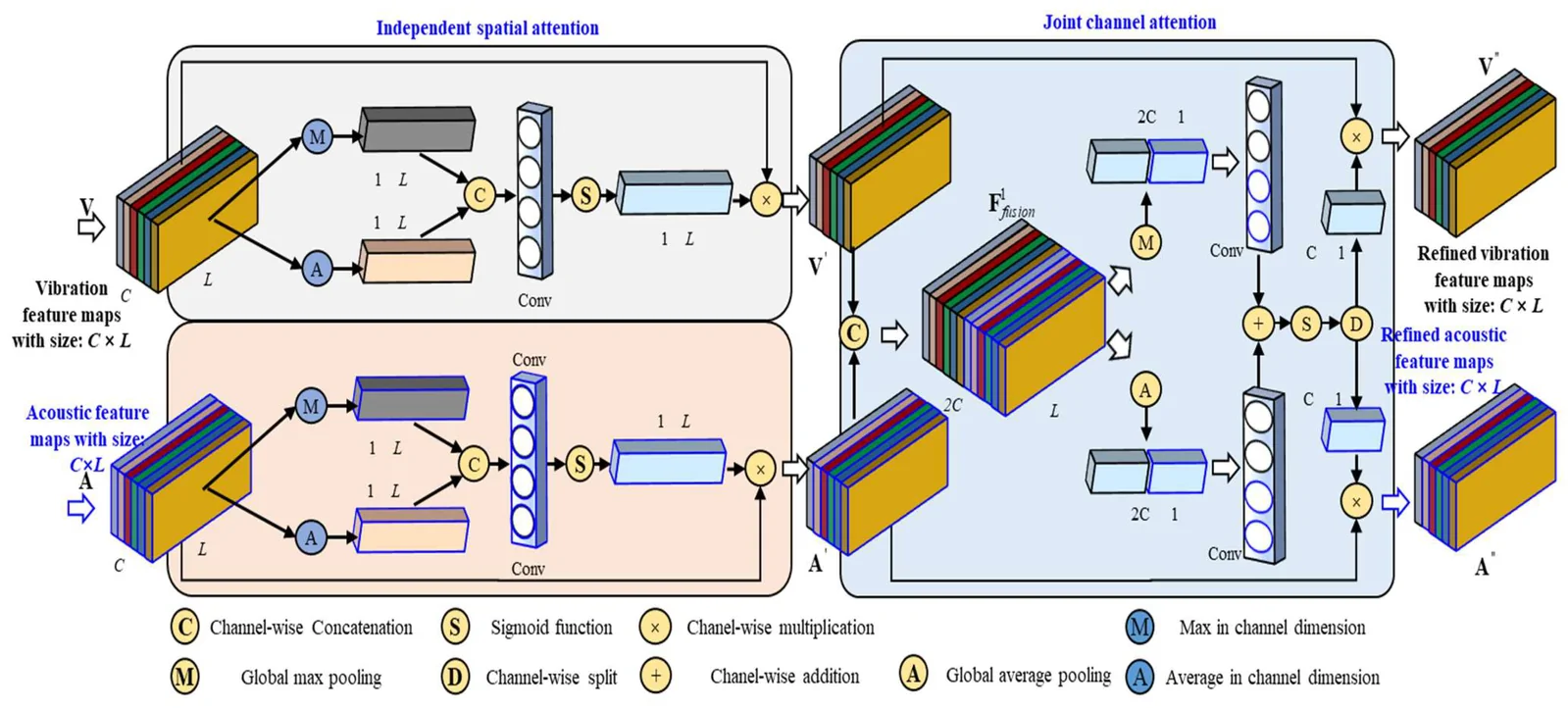
Multimodal cross-attention mechanism.

Vibration and acoustic measurements contain different amounts of fault information and also share cross-modal components. MCAM was designed to strengthen informative features within each modality and promote interaction between modalities. As shown in Figure 3, temporal attention is applied separately to the vibration and acoustic streams. The reweighted feature maps are concatenated along the channel dimension, and joint channel attention highlights fault-related channels while suppressing less informative components. The jointly weighted map is then split back into vibration and acoustic branches. The MCAM computations are defined as follows [30,35]:

(1) Let V and A denote the input vibration and acoustic feature maps. For each modality, maximum and mean pooling along the channel dimension compressed the feature map. The pooled maps were concatenated, convolved, and activated to obtain the reweighted outputs for V and A:

(2) $$V^{\prime }=f_{activation}\left(\mathrm{Conv}\left(\mathrm{Conc}\left[f_{\mathrm{Max}}\left(V\right),f_{Mean}\left(V\right)\right]\right)\right)⊗V$$

(3) $$A^{\prime }=f_{activation}\left(\mathrm{Conv}\left(\mathrm{Conc}\left[f_{\mathrm{Max}}\left(A\right),f_{Mean}\left(A\right)\right]\right)\right)⊗A$$

where $f_{\mathrm{Max}}$ and $f_{\mathrm{Mean}}$ denote channel-wise maximum and mean pooling, respectively; Conc denotes channel-wise concatenation; Conv and $f_{activation}$ denote convolution and activation; and ⊗ denotes channel-wise multiplication.

(2) The attended modality features $V^{\prime }$ and $A^{\prime }$ were concatenated along the channel dimension to obtain $F_{fusion}^{c}$. The fused feature map $F_{fusion}^{c}$ was passed to joint channel attention, which reweighted informative channels and suppressed irrelevant components. Specifically, $F_{fusion}^{c}$ underwent global maximum and average pooling, followed by convolution, channel-wise fusion, activation, and channel splitting. The resulting weights were multiplied element-wise with the original modality features to obtain $V^{\prime \prime }$ and $A^{\prime \prime }$:

(4) $$F_{fusion}^{c}=\mathrm{Conc}\left(V^{\prime },A^{\prime }\right)$$

(5) $$V^{\prime \prime }=Split_{V}\left(f_{activation}\left(\mathrm{Conv}\left(f_{MaxP}\left(F_{fusion}^{c}\right)\right)⊕\mathrm{Conv}\left(f_{\mathrm{AveP}}\left(F_{fusion}^{c}\right)\right)\right)\right)⊗V^{\prime }$$

(6) $$A^{\prime \prime }=Split_{A}\left(f_{activation}\left(\mathrm{Conv}\left(f_{MaxP}\left(F_{fusion}^{c}\right)\right)⊕\mathrm{Conv}\left(f_{\mathrm{AveP}}\left(F_{fusion}^{c}\right)\right)\right)\right)⊗A^{\prime }$$

where $f_{MaxP}$ and $f_{\mathrm{AveP}}$ denote global maximum and average pooling; $Split_{V}$ and $Split_{A}$ denote splitting the fused representation into the vibration $V^{\prime }$ and acoustic $A^{\prime }$ branches; and ⊕ denotes channel-wise addition.

#### 2.2.2. Cross-Modal Conditional-Decision Module

**Figure 4:**
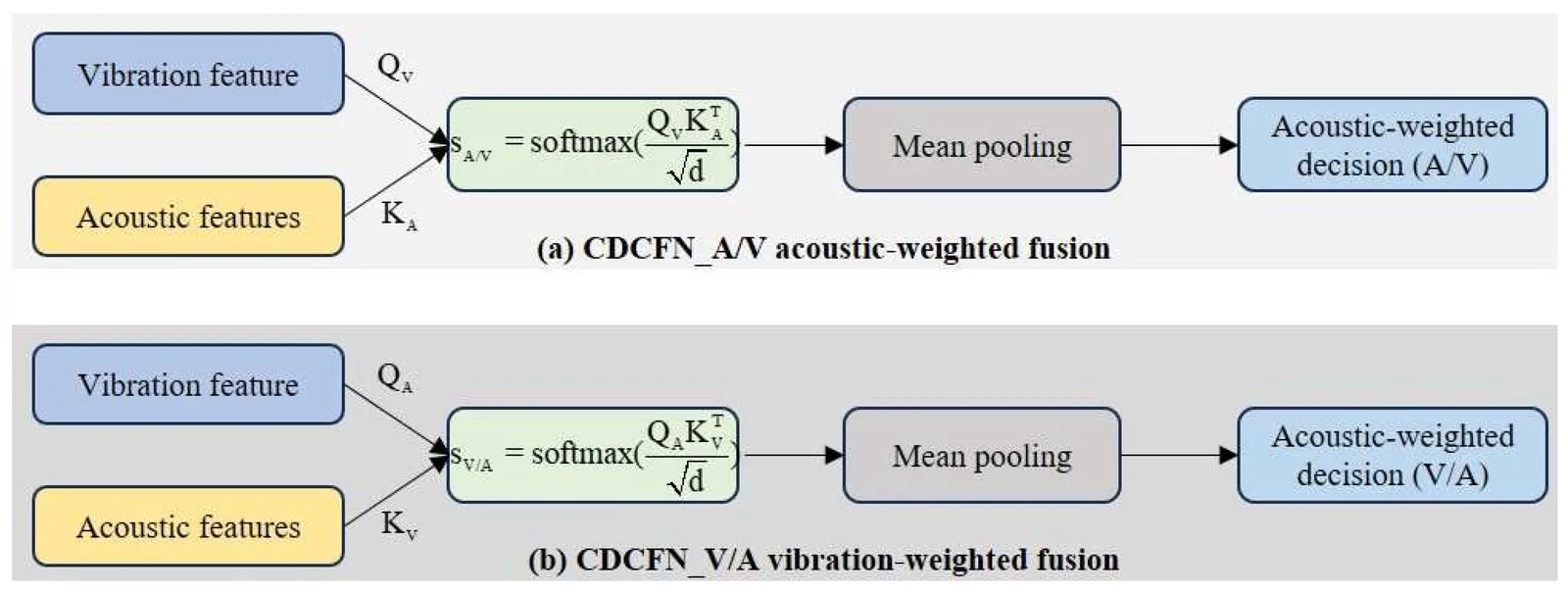
Implemented CMCD configurations: (**a**) CDCFN_A/V uses vibration queries and acoustic keys to form an acoustic-weighted decision vector. (**b**) CDCFN_V/A reverses the modalities to form a vibration-weighted decision vector. The classifier pools cross-modal attention scores; the value-weighted feature branch is not used for the final decision.

The acoustic and vibration streams follow different transmission paths and carry unequal fault information. CMCD therefore implements two directional cross-modal attention-score pooling operations, as shown in Figure 4. Unlike a conventional value-weighted cross-attention block [36], the implemented classifier pools the cross-modal attention scores to form a decision representation. The term ‘conditional’ describes the directional dependence of one modality’s decision weights on the other modality; it does not denote an explicitly estimated probability distribution.

Let $X_{V}$ and $X_{A}$ denote the vibration and acoustic feature sequences produced by the extraction module. The implemented CMCD uses one attention head. Linear projections generate the query and key representations required for the two reciprocal weighting directions.

For acoustic-weighted fusion (CDCFN_A/V), vibration provides $Q_{V}$ and acoustic features provide $K_{A}$. The attention-score matrix is $S_{A/V}=\mathrm{softmax}\left(\frac{Q_{V}K_{A}^{T}}{\sqrt{d}}\right)$. Mean pooling over query positions produces the acoustic-weighted decision vector.

For vibration-weighted fusion (CDCFN_V/A), acoustic features provide $Q_{A}$ and vibration features provide $K_{V}$. The reciprocal score matrix is $S_{V/A}=softmax\left(\frac{Q_{A}K_{V}^{T}}{\sqrt{d}}\right)$, and mean pooling over query positions produces the vibration-weighted decision vector.

The two variants therefore use the same CMCD operation with reversed query and key assignments. A/V denotes the acoustic-weighted decision guided by vibration, whereas V/A denotes the vibration-weighted decision guided by acoustic information.

This score-pooling implementation differs from standard cross-attention, which applies attention weights to a value matrix and returns an attended feature sequence. Here, the pooled cross-modal score pattern itself is passed to the final decision stage.

Accordingly, CMCD should be interpreted as reciprocal cross-modal attention weighting rather than explicit conditional-probability estimation.

CDCFN was implemented in PyTorch 1.10.0 and trained on an NVIDIA RTX 4080 GPU. The implemented model used one attention head and contained approximately 1.626 million trainable parameters for the 10-class task and 1.625 million for the 7-class task. Table 1 lists the network parameters.

CDCFN was trained for 100 epochs with a batch size of 16 using cross-entropy loss and stochastic gradient descent (momentum 0.9; weight decay 1 × 10^−4^). PyTorch’s default layer initialisation was used. The CyclicLR schedule used base_lr = 0.0001, max_lr = 0.1, triangular2 mode, and step_size_up = 2500 mini-batches; the triangular2 amplitude was halved after each complete cycle. No separate validation set or early-stopping rule was used. To avoid validation-based selection on this small dataset, the model after the fixed 100-epoch training schedule was evaluated on the predefined test set. The same optimiser and schedule were used for the comparison models.

## 3. Experimental Data Collection and Analysis

### 3.1. Engine Test Rig

**Figure 5:**
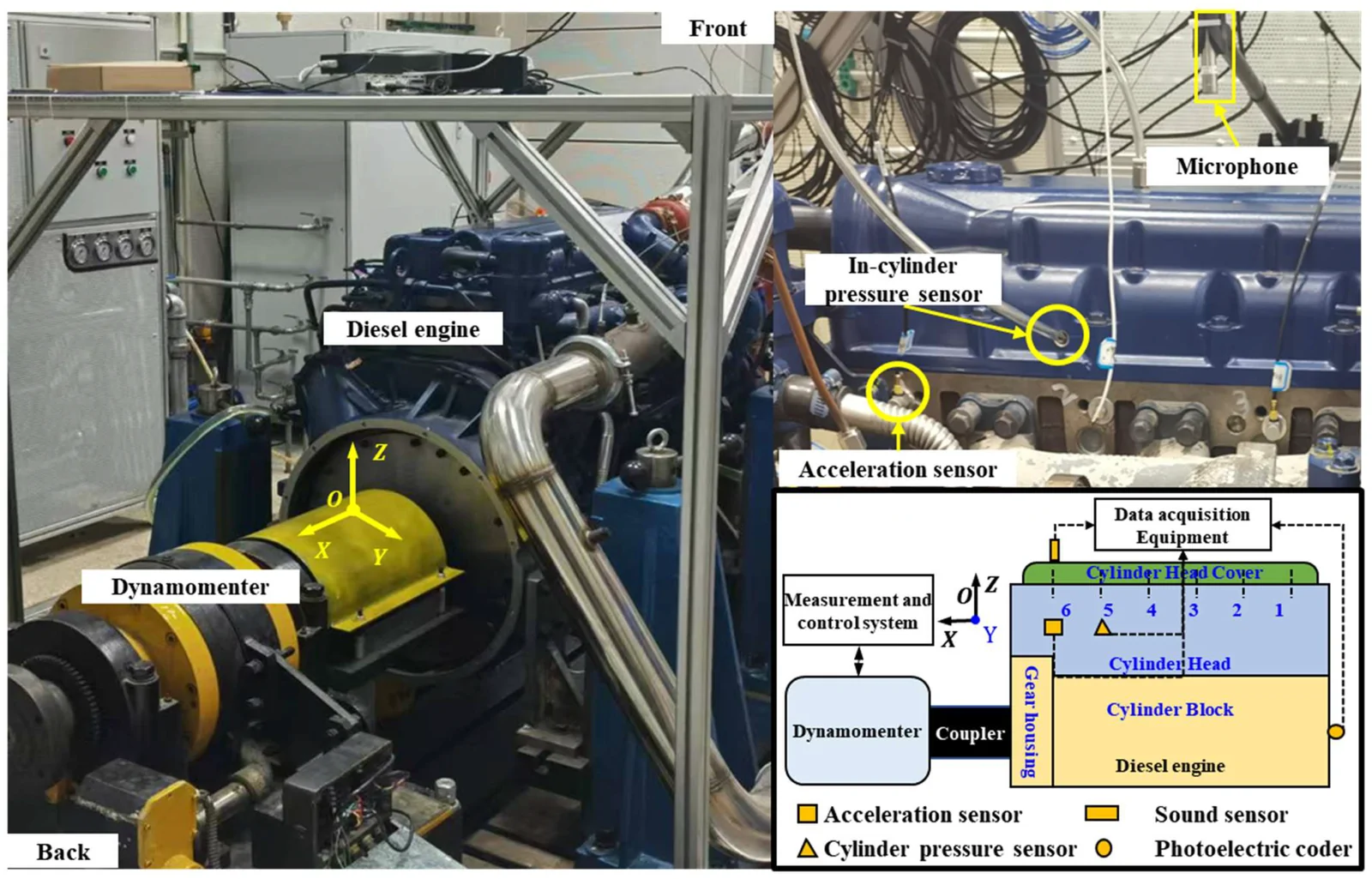
Diesel-engine test rig.

Vibro-acoustic experiments were conducted to measure cylinder-head vibration and radiated sound above cylinder 6. The test rig comprised a diesel engine, dynamometer, control system, and data-acquisition system (Figure 5). A 1A108E accelerometer (Donghua Testing Technology Co., Ltd., Jingjiang, China) was mounted on the exhaust side of cylinder 6’s head along the *y*-axis. A 6125C in-cylinder pressure sensor (Kistler Instrumente AG, Winterthur, Switzerland) was installed in cylinder 2. A 378B02 microphone (PCB Piezotronics, Inc., Depew, NY, USA) was positioned above cylinder 6 along the *z*-axis. An E6B2-CWZ3E photoelectric encoder (OMRON Corporation, Kyoto, Japan) measured the crankshaft angle.

Two datasets were collected to represent thermal faults (Dataset I) and mechanical faults (Dataset II). Thermal faults alter the intake charge and, consequently, affect combustion, power, fuel efficiency, and emissions. Mechanical faults represent defects in critical components and can reduce operational reliability. All tests were conducted at 1200 r/min under four throttle openings (0%, 25%, 50%, and 75%; Table 2). Cylinder-pressure, vibration, and acoustic signals were sampled at 51.2 kHz. One 30 s continuous record was acquired for each fault-class–load combination, and a 15 s segment was used in the present analysis.

### 3.2. Consistency of Vibro-Acoustic Responses with the Engine Cycle

**Figure 6:**
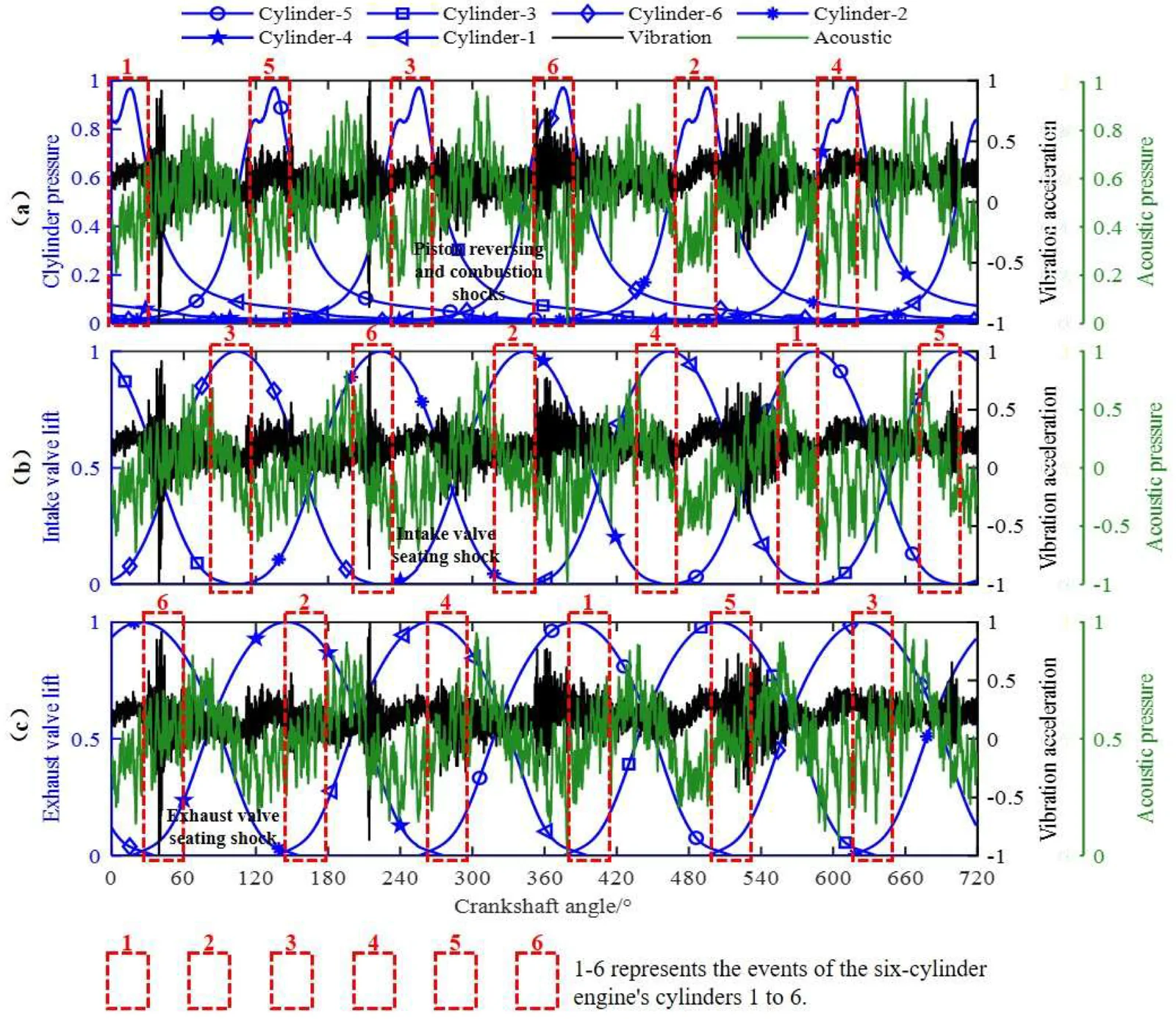
Correspondence between engine vibro-acoustic responses and the thermodynamic cycle under normal operation: (**a**) Cylinder pressure and vibro-acoustic signal, (**b**) Intake valve lift and vibro-acoustic signal and (**c**) Exhaust valve lift and vibro-acoustic signal.

Each cylinder undergoes intake, compression, combustion, and exhaust during one engine cycle. The firing order is 1-5-3-6-2-4, with successive cylinders separated by 120 degrees of crank angle. Surface-response signals therefore contain periodic components associated with these processes [25,37]. Figure 6 compares the crank-angle-domain vibro-acoustic responses with in-cylinder pressure and valve-lift curves under normal operation. Vibration and acoustic signals were normalised to [−1, 1], whereas pressure and valve-lift signals were normalised to [0, 1].

Figure 6a compares in-cylinder pressure with the vibro-acoustic signals. Combustion occurs near top dead centre and produces a rapid pressure rise followed by a gradual decay. The vibration signal consequently contains strong responses around 360 degrees from piston reversal and combustion shock (PRS and CS). Radiated sound loses energy during transmission through the engine structure and air. Its impact components are less distinct, but its periodicity remains consistent with the combustion frequency of the six-cylinder engine. Figure 6b compares the intake-valve-lift curve with the vibro-acoustic signals. Each intake valve opens and closes once per cylinder cycle. Closure at approximately 210 degrees produces a distinct vibration response attributed to intake-valve seating shock (IVS). Figure 6c compares the exhaust-valve-lift curve with the vibro-acoustic signals. Each exhaust valve also opens and closes once per cylinder cycle. Closure at approximately 40 degrees produces a distinct vibration response attributed to exhaust-valve seating shock (EVS).

The measured vibro-acoustic signals therefore capture exhaust-valve seating, intake-valve seating, piston reversal, and combustion responses. Because the six cylinders operate sequentially at 120-degree intervals, their responses are individually generated but superimposed in the surface measurements. This superposition distinguishes reciprocating engines from simpler rotating components such as bearings and gears and increases the difficulty of fault diagnosis.

### 3.3. Dataset I: Thermal Faults

**Figure 7:**
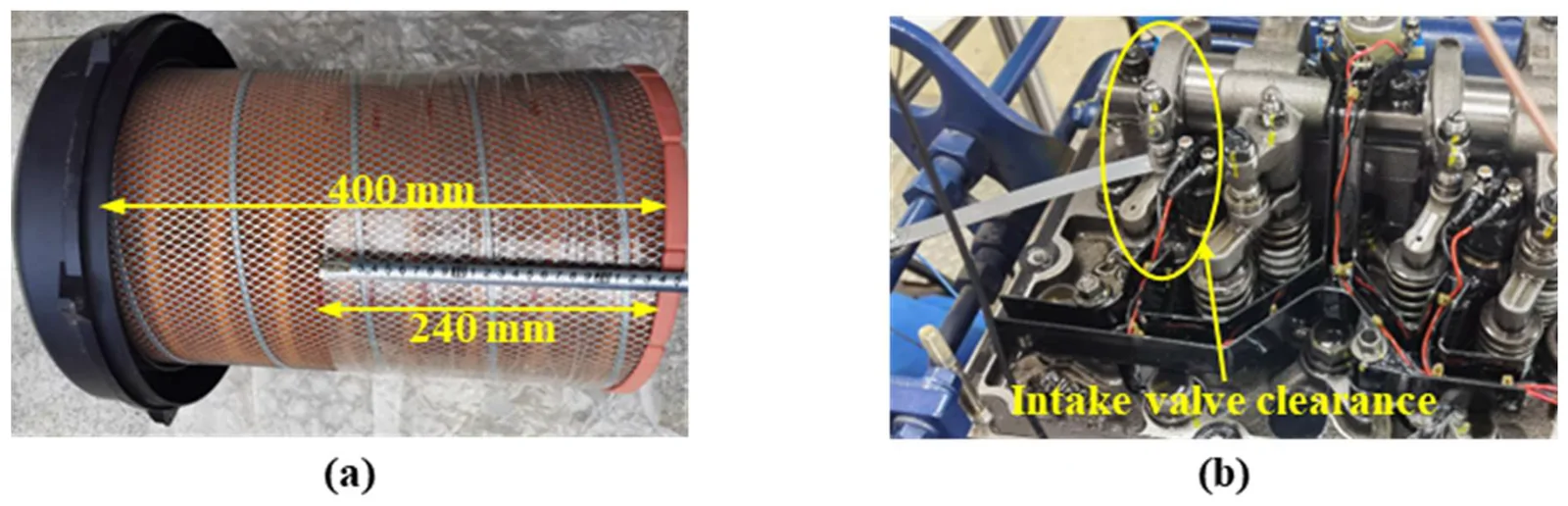
Thermal faults: (**a**) 60% intake-filter blockage; (**b**) abnormal intake-valve clearance.

Figure 7 shows the thermal-fault configurations. The intake-filter blockage in Figure 7a reduced the total intake charge by obstructing a specified proportion of the filter element. The abnormal intake-valve clearance in Figure 7b reduced the intake volume of one cylinder by adjusting the clearance with a piston ruler and stud. Both faults degraded combustion and engine power. Table 3 lists the fault settings. For each fault-class–load combination, the selected 15 s vibration and acoustic records were synchronously converted to the angle domain and divided into 150 non-overlapping paired windows (5120 points per modality; stride 5120 points). The paired samples were randomly ordered, after which 50 pairs per class were assigned to training and 100 to testing. Thus, the 150 pairs comprise 150 vibration and 150 acoustic single-modality samples. The training and test windows did not overlap, but they originated from the same continuous acquisition.

**Figure 8:**
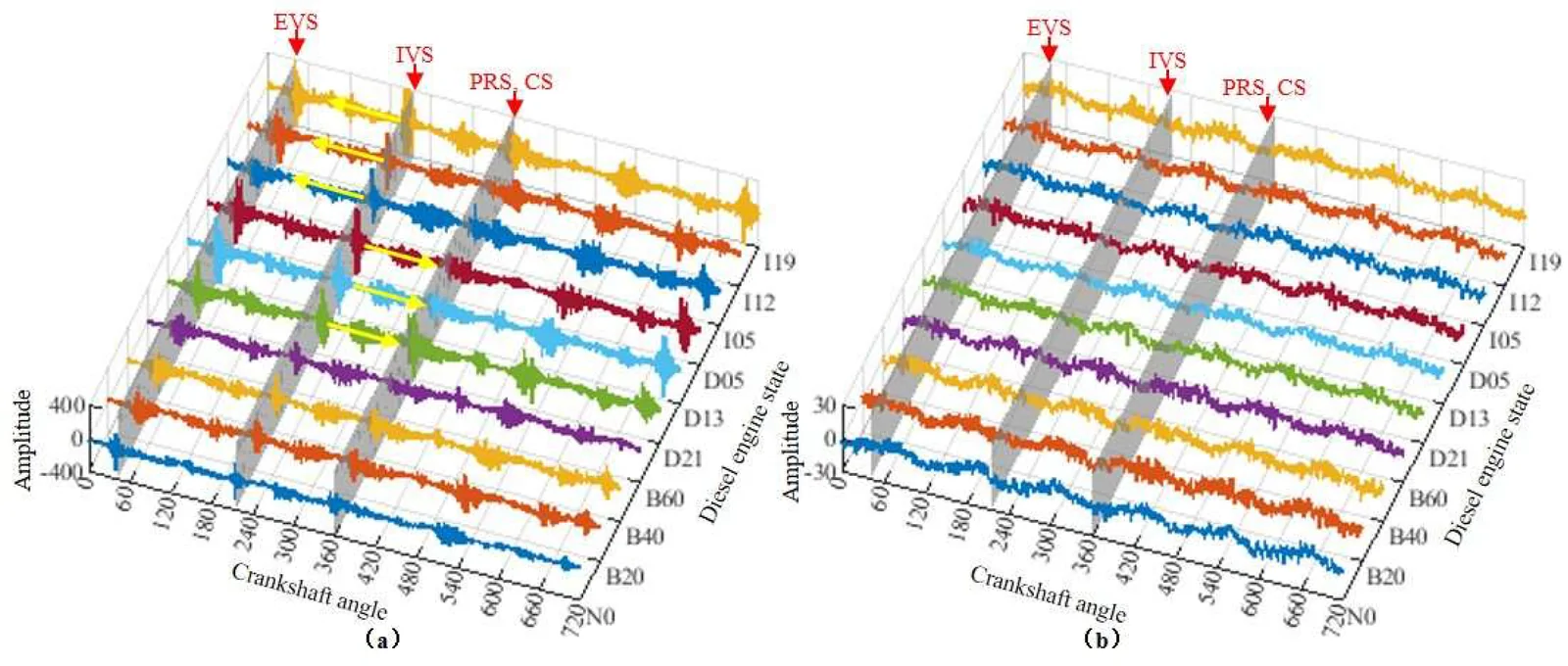
Vibro-acoustic signals for thermal faults: (**a**) vibration signal; (**b**) acoustic signal.

Figure 8 shows the vibration and acoustic signals for the thermal faults in Table 3. The vibration signals contain distinct EVS, IVS, PRS, and CS responses aligned with the engine cycle. Intake-filter blockage reduces the intake charge of all cylinders and mainly changes the combustion response. Abnormal intake-valve clearance affects one cylinder and changes both its intake volume and valve timing, producing an advance or delay in IVS. The acoustic signals contain fewer distinct impact components, making the individual operating states more difficult to separate by visual inspection.

### 3.4. Dataset II: Mechanical Faults

**Figure 9:**
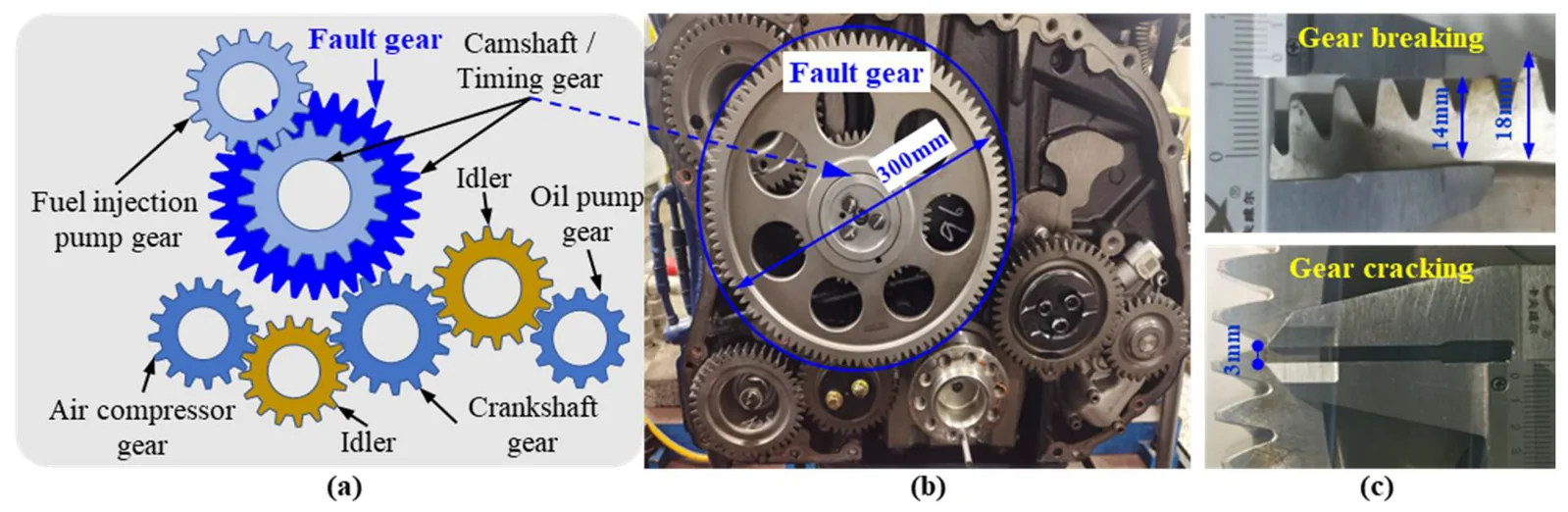
Mechanical-fault configurations: (**a**) rear timing-gear train; (**b**) physical gear-train layout; (**c**) cracked and broken-tooth timing gears.

Figure 9 shows the engine timing-gear train and the damaged components. The rear gear train contains the crankshaft, camshaft-timing, fuel-injection-pump, air-compressor, and oil-pump gears (Figure 9a,b). Mechanical faults were introduced as cracks and broken teeth in the camshaft-timing gear (Figure 9c). Besides transmitting motion and torque, the timing gear coordinates fuel injection and valve opening and closure. Its failure can therefore disturb both combustion and mechanical stability. Table 4 lists the mechanical-fault settings. Dataset II used the same preprocessing and partitioning protocol as Dataset I: one 15 s segment from a single continuous acquisition for each fault-class–load combination was synchronously converted to the angle domain and divided into 150 non-overlapping paired windows of 5120 points (stride 5120). After random ordering, 50 paired samples per class were used for training and 100 for testing. The two sets therefore contained distinct windows from the same acquisition, rather than independent experimental runs.

**Figure 10:**
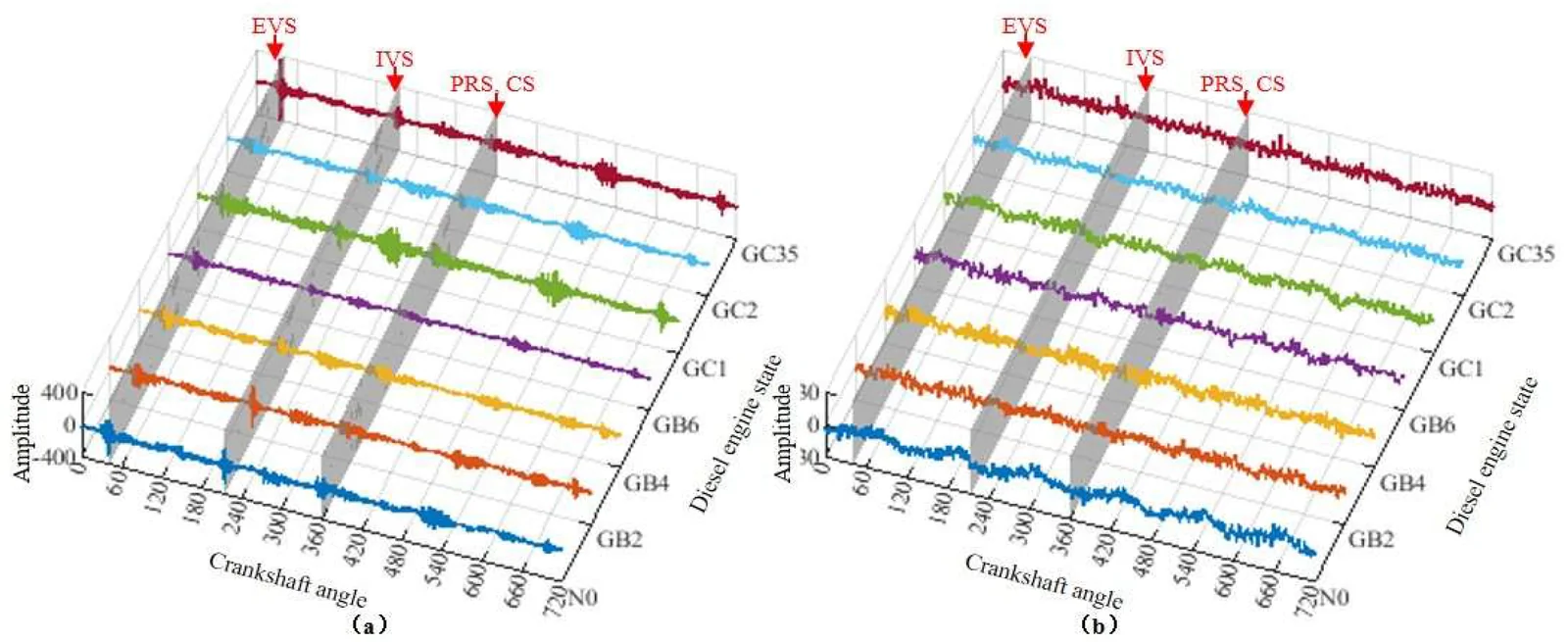
Vibro-acoustic signals for mechanical faults: (**a**) vibration signal; (**b**) acoustic signal.

Figure 10 shows the vibration and acoustic signals for the mechanical faults in Table 4. The vibration signals retain distinct EVS, IVS, PRS, and CS components under all conditions. Timing-gear faults alter the overall vibration energy and combustion timing, but they do not produce the clear valve-event advance or delay observed for thermal faults. The acoustic signals again contain less distinct impact components, making the operating states difficult to distinguish directly.

## 4. Results and Discussion

### 4.1. Ablation Analysis

Five metrics were used to evaluate classification performance: accuracy, precision, recall, F1 score, and Cohen’s kappa [38].

(1) Accuracy is the proportion of all samples assigned to the correct class. It summarises overall classification performance but does not distinguish among types of error:

(7) $$Acurracy=\frac{TP+TN}{TP+FP+TN+FN}$$

(2) Precision is the proportion of predicted positive samples that are true positives. It quantifies the reliability of positive predictions:

(8) $$Precision=\frac{TP}{TP+FP}$$

(3) Recall is the proportion of actual positive samples that are correctly identified. It quantifies the completeness of positive-class detection:

(9) $$Recall=\frac{TP}{TP+FN}$$

(4) The F1 score is the harmonic mean of precision and recall. Values closer to 1 indicate a stronger balance between the two metrics:

(10) $$F1-Score=\frac{2\times Precision\times Recall}{Precision+Recall}$$

(5) Cohen’s kappa measures agreement between predictions and reference labels after accounting for chance agreement. Values range from −1 to 1, with higher values indicating stronger agreement:

(11) $$Kappa=\frac{p_{o}-p_{e}}{1-p_{e}}$$

where $p_{o}=\left(TP+TN\right)/N$ is the observed accuracy obtained from the diagonal of the confusion matrix, and $p_{e}=\left[\left(TP+FN\right)\cdot \left(TP+FP\right)+\left(FP+TN\right)\cdot \left(FN+TN\right)\right]/N^{2}$ is the expected agreement by chance.

True positives (TP) and true negatives (TN) are correctly classified positive and negative samples, respectively. False negatives (FN) are positive samples classified as negative, whereas false positives (FP) are negative samples classified as positive. Lower FN and FP values therefore indicate fewer missed detections and false alarms. Precision, recall and F1 score were calculated as class-frequency-weighted metrics. Each experiment was repeated ten times with independent model initialisation, and the mean and standard deviation of each metric were reported.

**Figure 11:**
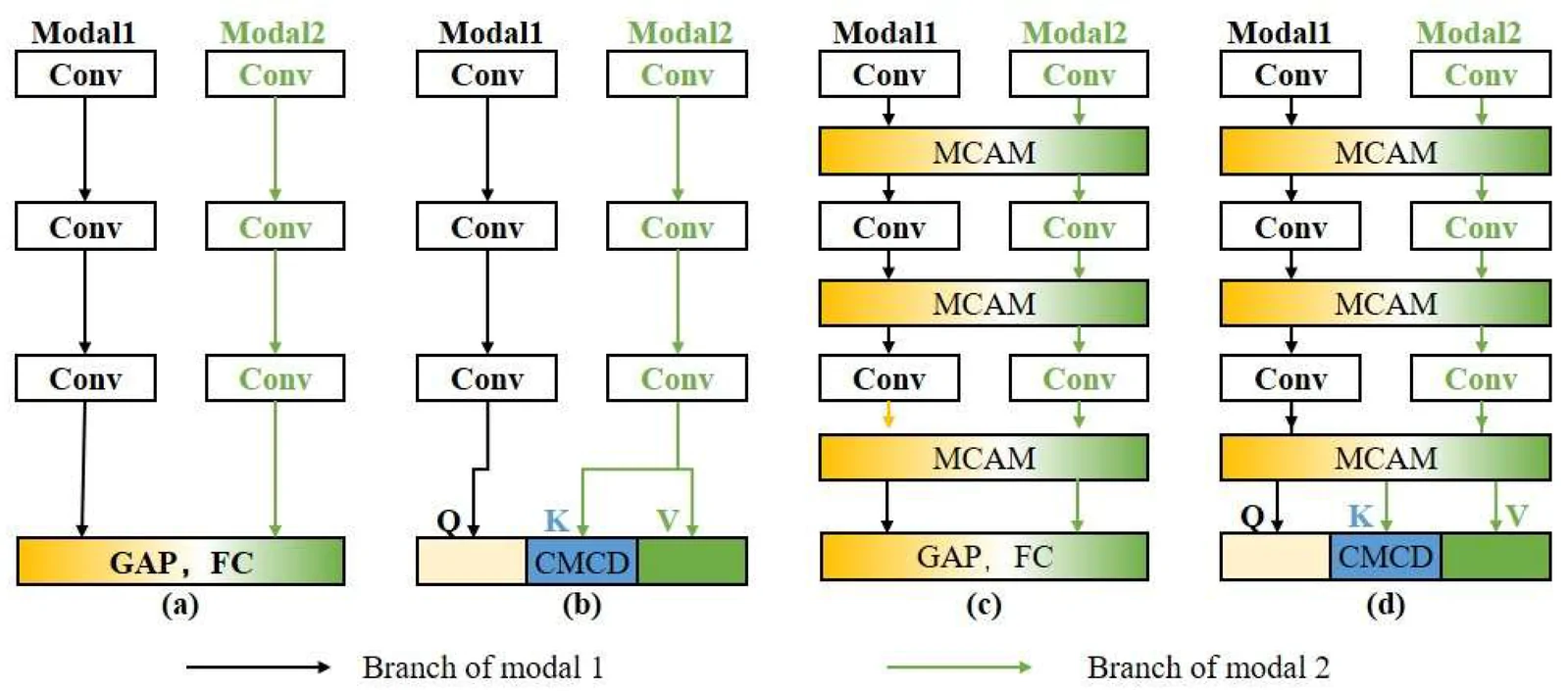
Architectures used in the ablation study: (**a**) MCNN; (**b**) MCNNCD; (**c**) DCFN; (**d**) CDCFN.

Six variants from four network architectures were compared in the ablation analysis (Figure 11). MCNN is a conventional CNN-based fusion network. MCNNCD replaces its classifier with CMCD, and DCFN adds MCAM to the CNN feature streams. CDCFN contains both MCAM and CMCD. In MCAM, each modality first undergoes temporal attention; the attended maps are concatenated for joint channel attention and then separated. CMCD supports two reciprocal weighting directions: CDCFN_A/V denotes an acoustic-weighted decision guided by vibration, whereas CDCFN_V/A denotes a vibration-weighted decision guided by acoustic information. All variants were trained with identical hyperparameters.

#### 4.1.1. Analysis Result for Dataset I: Thermal Faults

**Figure 12:**
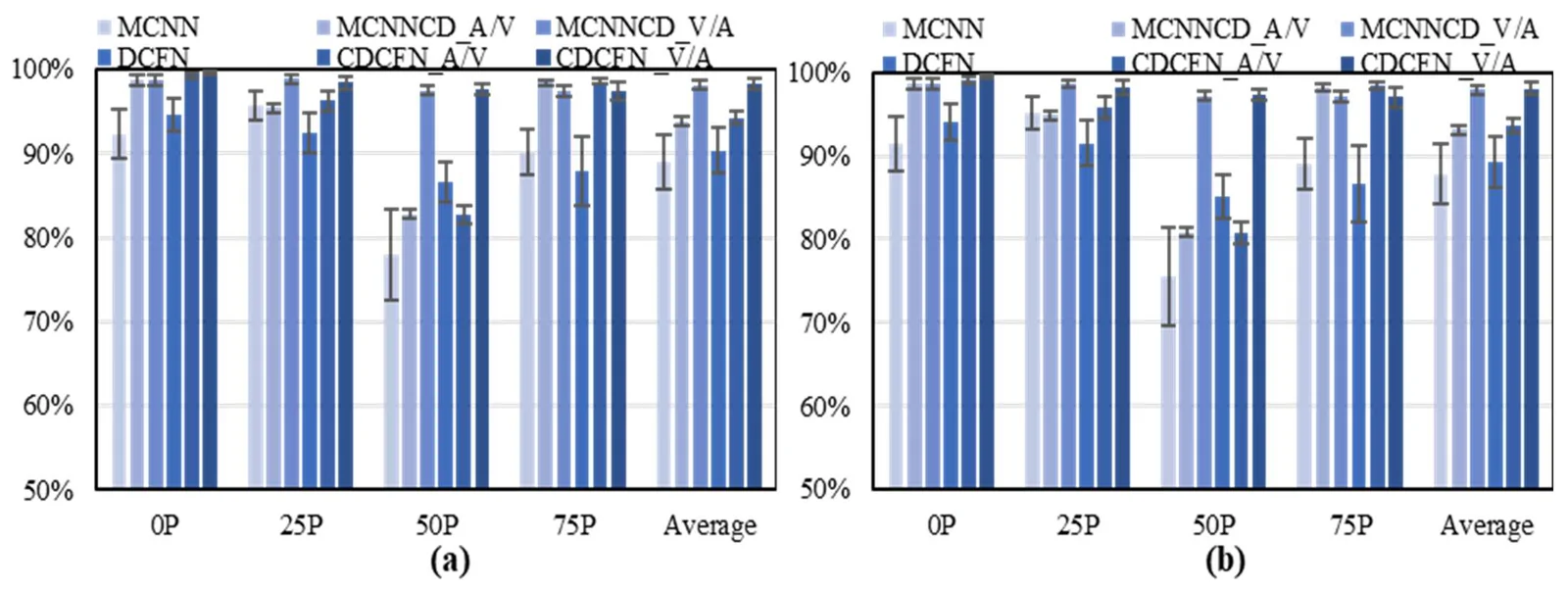
Recognition results for Dataset I: (**a**) accuracy; (**b**) Cohen’s kappa.

The six model variants were first evaluated on the thermal-fault dataset (Figure 12 and Table 5). Accuracy varied nonlinearly with load, and no single ordering was preserved at every load. Averaged across the four tested loads, CDCFN_A/V and CDCFN_V/A achieved 94.18 ± 0.78% and 98.23 ± 0.68% accuracy, respectively. DCFN achieved 90.37 ± 2.71%, MCNNCD_V/A and MCNNCD_A/V achieved 98.10 ± 0.56% and 93.82 ± 0.53%, respectively, and MCNN achieved 89.02 ± 3.23%. The 0.13-percentage-point difference between CDCFN_V/A and MCNNCD_V/A is small relative to the reported run-to-run variation and is therefore described as a numerical, rather than statistically established, improvement. The results indicate that the preferred weighting direction depends on the dataset and operating condition.

#### 4.1.2. Analysis Result for Dataset II: Mechanical Faults

**Figure 13:**
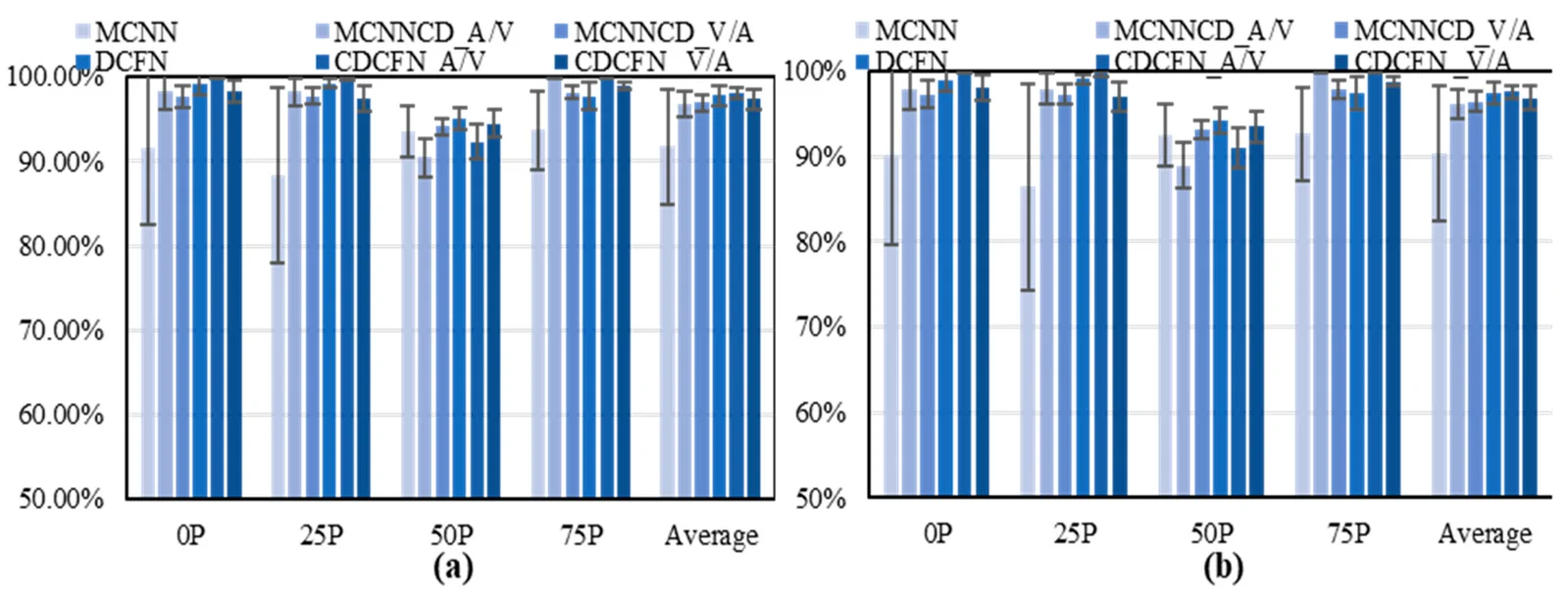
Recognition results for Dataset II: (**a**) accuracy; (**b**) Cohen’s kappa.

The same six variants were evaluated on the mechanical-fault dataset (Figure 13 and Table 6). Accuracy again fluctuated with load. Averaged across the four tested loads, CDCFN_A/V and CDCFN_V/A achieved 97.99 ± 0.63% and 97.33 ± 1.22% accuracy, respectively. DCFN achieved 97.81%, MCNNCD_V/A and MCNNCD_A/V achieved 96.93% and 96.73%, respectively, and MCNN achieved 91.79%. The kappa values in Figure 13b showed a similar overall pattern. The close A/V and V/A results indicate that the two directional variants provided comparable performance for mechanical faults.

Together, Figure 13 and Table 6 show that MCAM and CMCD supported high recognition of mechanical faults across the four tested load conditions. Because all loads were represented in both the training and test sets, these results do not constitute leave-one-load-out generalisation.

### 4.2. Noise Resistance

Noise resistance was evaluated by adding zero-mean Gaussian white noise to the segmented angle-domain time-series samples before network input. Noise was generated independently for the vibration and acoustic modalities and independently for the training and test sets. SNRs of −12, −8, −4, 0, 4, 8, and 12 dB were evaluated. For each SNR, every model was retrained from scratch and evaluated under the same SNR, constituting matched-noise training and testing rather than clean-to-noisy generalisation. Ten repeated runs were performed with fresh noise realisations. SNR was calculated from signal and noise energy as follows:

(12) $$SNR=10\log\frac{P_{signal}}{P_{noise}}$$

where $P_{signal}$ denotes the energy of the measured signal and $P_{noise}$ denotes noise energy.

#### 4.2.1. Anti-Noise Analysis for Dataset I: Thermal Faults

**Figure 14:**
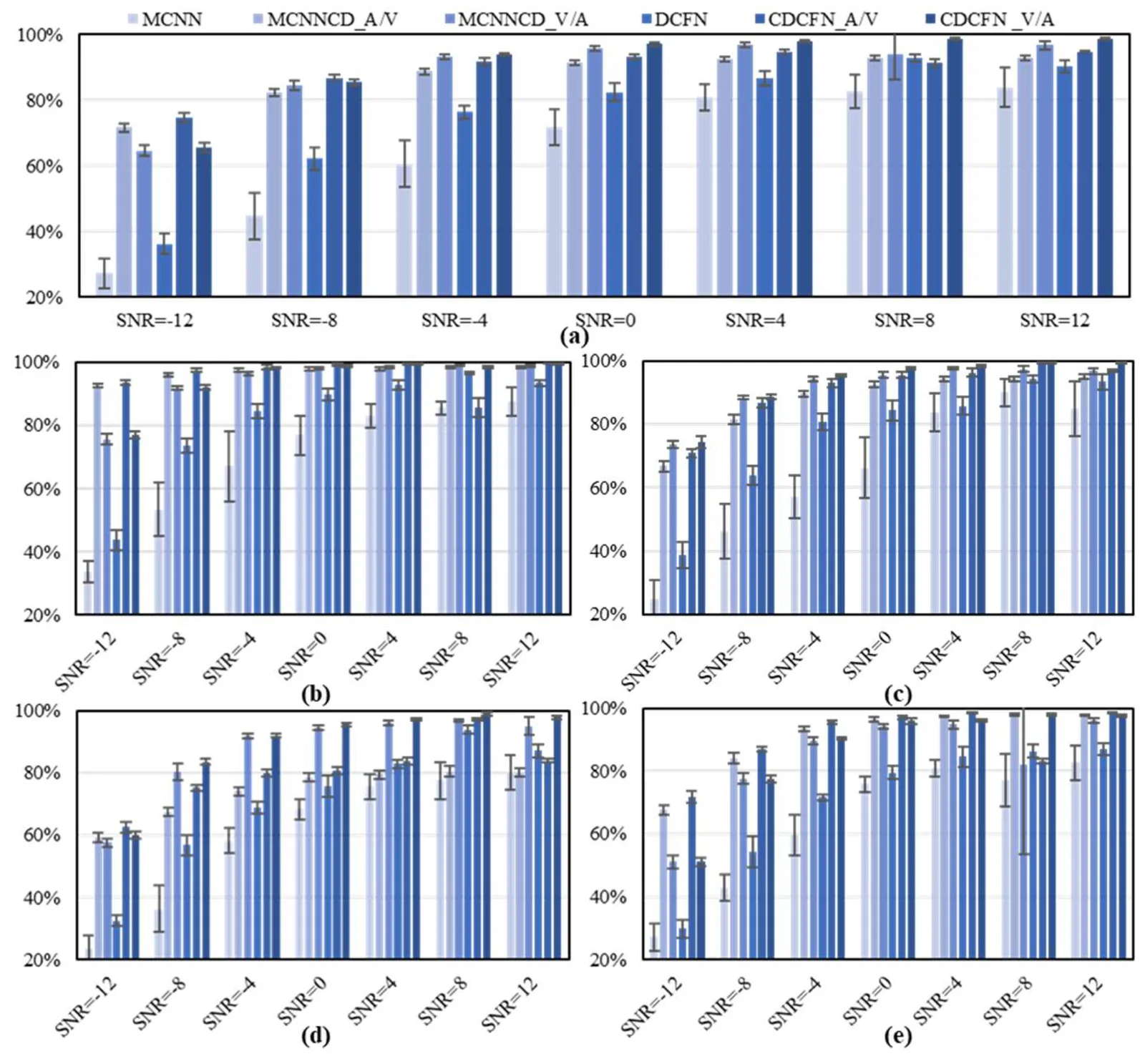
Mean recognition accuracy and standard deviation for Dataset I under added noise: (**a**) average across loads; (**b**) 0% load; (**c**) 25% load; (**d**) 50% load; (**e**) 75% load.

The four network architectures were evaluated on Dataset I at SNRs of −12, −8, −4, 0, 4, 8, and 12 dB (Figure 14). Accuracy generally increased with SNR but varied with load and weighting direction. At −12 dB, the across-load mean accuracies of CDCFN_A/V and CDCFN_V/A were 94.89 ± 0.34% and 54.61 ± 1.65%, respectively. The marked directional difference indicates that acoustic-weighted decisions guided by vibration were more effective for this dataset under the strongest matched-noise condition. These results quantify performance only when training and testing use the same SNR.

Across the tested matched-noise settings, CDCFN_A/V retained comparatively high thermal-fault recognition at low SNR. Unequal SNRs between the two modalities and clean-to-noisy transfer were not evaluated.

#### 4.2.2. Anti-Noise Analysis for Dataset II: Mechanical Faults

**Figure 15:**
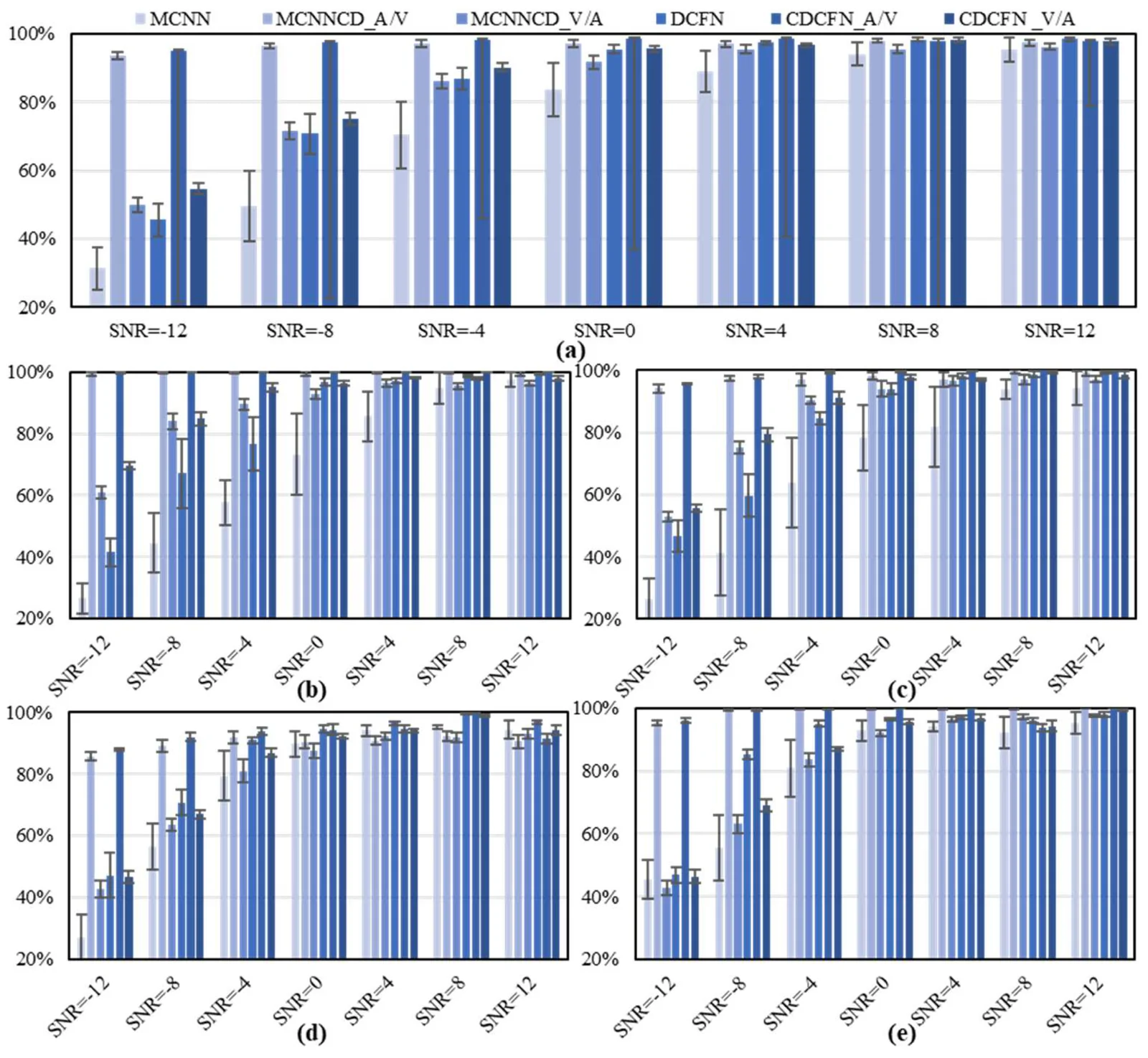
Mean recognition accuracy and standard deviation for Dataset II under added noise: (**a**) average across loads; (**b**) 0% load; (**c**) 25% load; (**d**) 50% load; (**e**) 75% load.

The four architectures were also evaluated on Dataset II at SNRs of −12, −8, −4, 0, 4, 8, and 12 dB (Figure 15). At −12 dB, the across-load mean accuracies of CDCFN_A/V and CDCFN_V/A were 74.71 ± 1.45% and 65.60 ± 1.46%, respectively. Accuracy generally improved with SNR but varied across loads, and the A/V direction was more accurate under the strongest matched-noise condition. The previously quoted values of 99.91% and 69.63% referred to individual load-specific results and are not used as across-load summaries.

For mechanical faults, CDCFN_A/V provided the higher across-load mean accuracy at −12 dB under matched-noise training and testing. This experiment does not establish robustness when the modalities have unequal noise levels or when a clean-trained model is tested on noisy data.

### 4.3. Computational Efficiency

Computational efficiency was assessed for the four ablation architectures on Dataset I using the hardware and software environment described in Section 2.2.2. Each model was trained for 100 epochs on 500 training samples and evaluated on 1000 test samples. Wall-clock training and testing times were measured over five independent executions and are reported as means ± standard deviations (shown in Table 7). The test time was additionally divided by the number of test samples to obtain the mean per-sample inference time.

CDCFN required 186.69 ± 1.81 s for training and 0.749 ± 0.018 s to process 1000 test samples, or approximately 0.749 ms per sample. Its training time was comparable to that of DCFN; its test time was lower than that of DCFN but higher than those of MCNN and MCNNCD. Thus, the additional attention operations increased runtime relative to MCNN, although GPU inference remained below 1 ms per sample. These measurements are implementation-specific; memory use and CPU latency were not profiled, so no platform-independent efficiency claim is made here.

### 4.4. Comparison with Existing Methods

CDCFN was compared with six existing fusion architectures: MJA-Net [30] uses a convolutional block attention module for vibro-acoustic fusion and a fully connected classifier. SMJA-Net and EMJA-Net replace that module with squeeze-and-excitation and external channel attention, respectively. The remaining models were a non-local neural network (NLNN) [39], a Vision Transformer (VisionT) using one MJA-Net fusion configuration [40], and a dual-branch cross-modal CNN without attention (CMCNN) [41].

All models were trained with the same predefined optimiser and learning-rate schedule. Accuracy, weighted precision, weighted recall, weighted F1 score, and Cohen’s kappa were used to compare recognition performance on both datasets. Section 4.3 reports the measured training and test times of the four ablation architectures. For the six external benchmark architectures, matched runtime implementations were not available; their comparison is therefore limited to predictive performance under the common data protocol.

#### 4.4.1. Comparison for Dataset I: Thermal Faults

**Figure 16:**
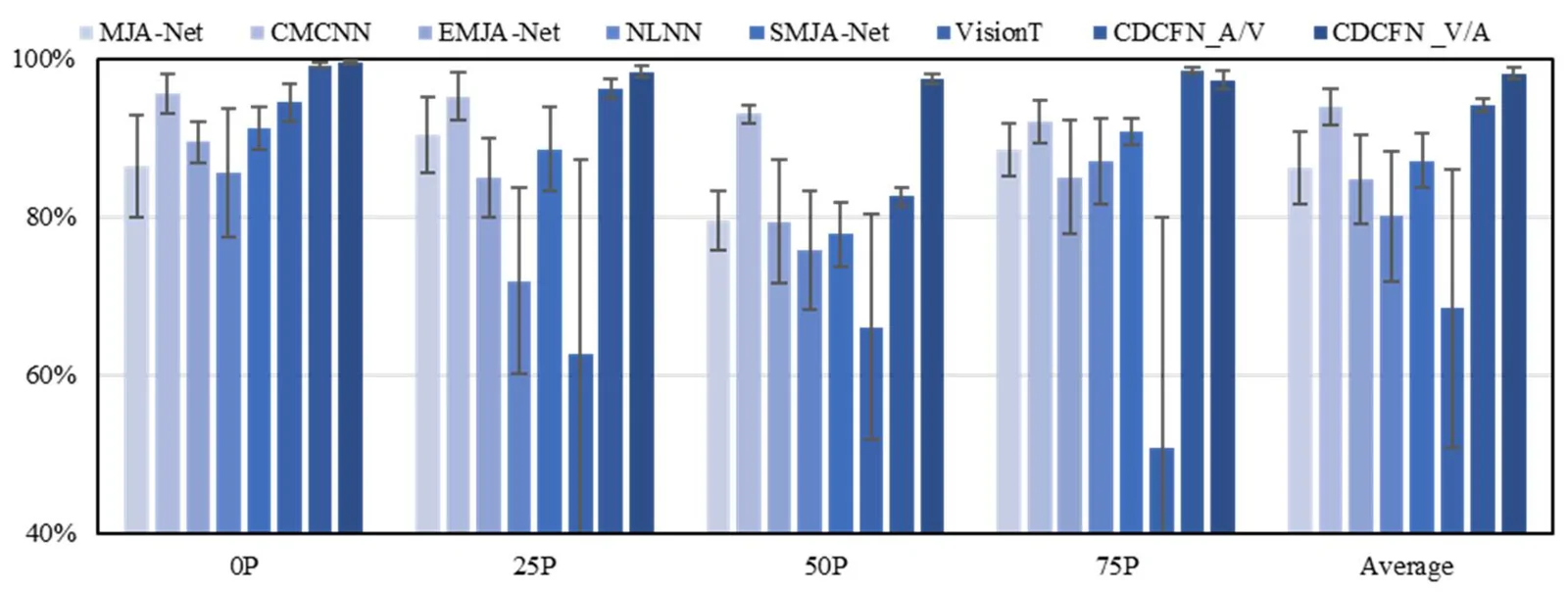
Recognition accuracy for Dataset I across comparison models.

Figure 16 compares mean accuracy and standard deviation on Dataset I. At 0% load, CDCFN_A/V and CDCFN_V/A achieved 99.23 ± 0.37% and 99.59 ± 0.31%, respectively. MJA-Net, SMJA-Net, and EMJA-Net achieved 86.42 ± 6.49%, 91.27 ± 2.65%, and 89.58 ± 2.61%, whereas NLNN, VisionT, and CMCNN achieved 85.62 ± 8.11%, 94.50 ± 2.41%, and 95.62 ± 2.48%, respectively. Accuracy varied with load and directional configuration. Averaged across the four tested loads, CDCFN_A/V and CDCFN_V/A achieved 94.18 ± 0.78% and 98.23 ± 0.68%, respectively. These values indicate higher numerical performance for CDCFN_V/A within the evaluated protocol, but they do not demonstrate transfer to an unseen load.

#### 4.4.2. Comparison for Dataset II: Mechanical Faults

**Figure 17:**
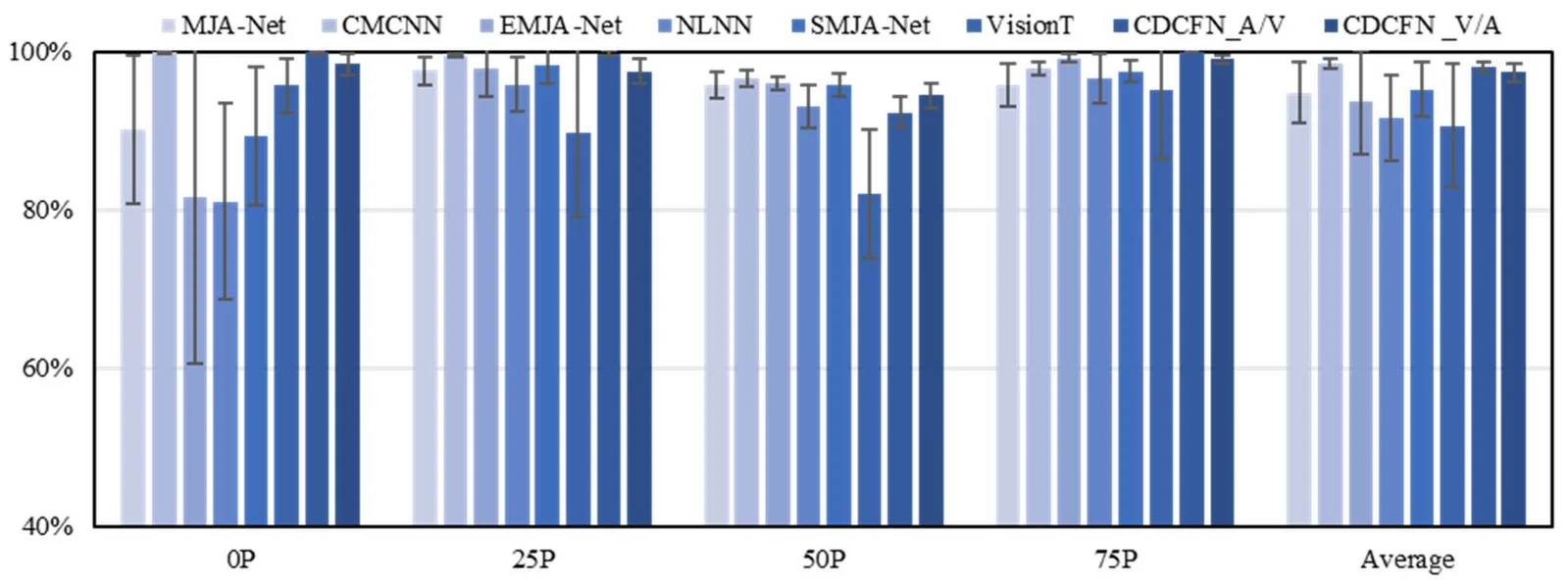
Recognition accuracy for Dataset II across comparison models.

Figure 17 compares mean accuracy and standard deviation on Dataset II. At 0% load, CDCFN_A/V and CDCFN_V/A achieved 99.91 ± 0.15% and 98.36 ± 1.31%, respectively. MJA-Net, SMJA-Net, and EMJA-Net achieved 90.14 ± 9.42%, 89.37 ± 8.77%, and 81.59 ± 21.01%, whereas NLNN, VisionT, and CMCNN achieved 81.03 ± 12.39%, 95.69 ± 3.41%, and 99.87 ± 0.26%, respectively. Accuracy fluctuated with load, particularly at 50%. Averaged across the four tested loads, CDCFN_A/V and CDCFN_V/A achieved 97.99 ± 0.63% and 97.33 ± 1.22%, respectively. The small differences among the leading models should be interpreted in relation to the reported run-to-run variation.

Figure 16 and Figure 17 show that CDCFN achieved high mean accuracy with comparatively low variability on both datasets under the evaluated within-load protocol. The results support performance across the tested load conditions, not leave-one-load-out generalisation.

### 4.5. Feature Visualisation with LayerCAM

LayerCAM [42] was used to identify the signal segments contributing most strongly to CDCFN decisions. Activations were computed from the vibro-acoustic features in the final convolutional layer of Block 3, upsampled and mapped to the original signals. Higher activation values indicate a larger contribution to the predicted class. The maps were interpreted against the engine operating cycle to assess whether the learned features were physically plausible.

#### 4.5.1. LayerCAM Analysis for Dataset I: Thermal Faults

**Figure 18:**
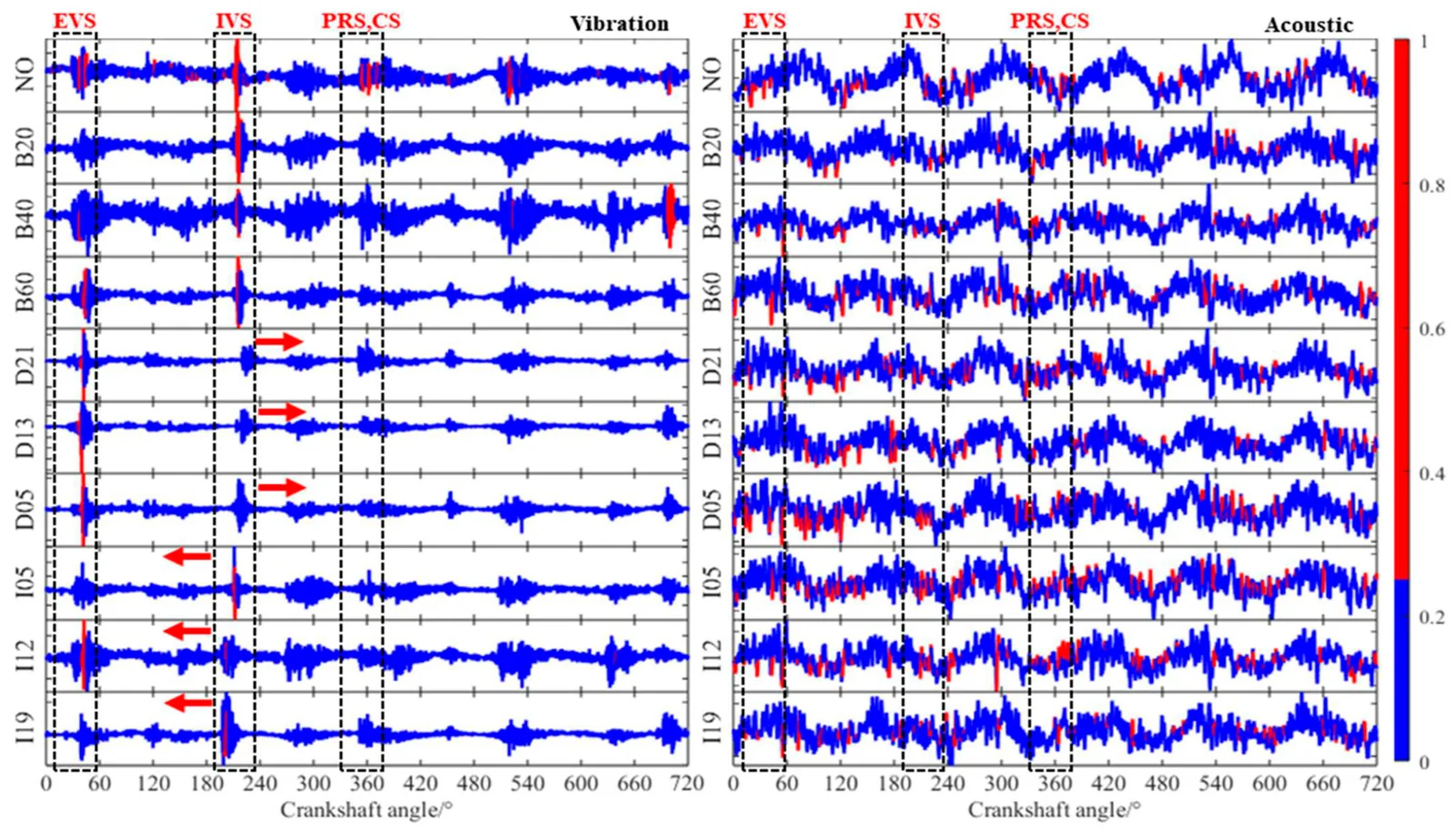
LayerCAM visualisations of CDCFN_A/V for Dataset I.

**Figure 19:**
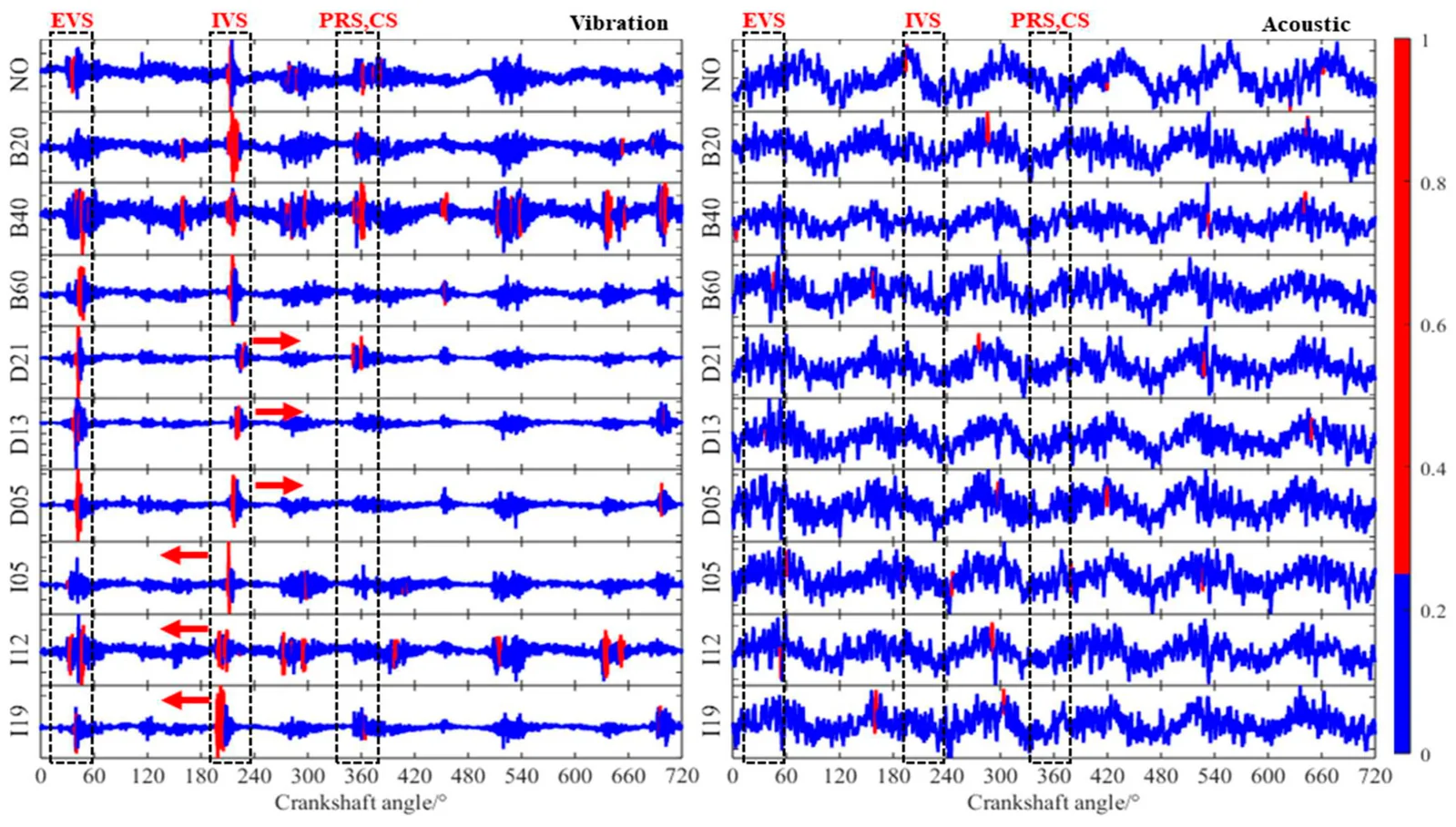
LayerCAM visualisations of CDCFN_V/A for Dataset I.

At 1200 RPM and 50% load, CDCFN_A/V and CDCFN_V/A achieved 87.29% and 98.30% accuracy on Dataset I, respectively. Figure 18 and Figure 19 show their activation maps. Intake-filter blockage changes the total air intake of all six cylinders, whereas abnormal intake-valve clearance changes the intake of one cylinder. Both faults consequently affect combustion and exhaust. Vibration signals contain distinct impact components and correspondingly concentrated activations. Acoustic signals contain fewer visible impact components and more diffuse activations.

Figure 18 shows that CDCFN_A/V attended to EVS, IVS, PRS, and CS in the vibration signals. Under normal conditions, all four components received high attention. IVS dominated for intake-filter blockage, whereas EVS dominated for abnormal valve clearance. Relative to the operating-cycle analysis in Figure 6, CDCFN_A/V captured the principal engine impacts but placed less emphasis on fault-specific components, consistent with its lower accuracy.

Figure 19 shows that CDCFN_V/A also attended to EVS, IVS, PRS, and CS. Under normal conditions, all four components received high attention. IVS dominated for intake-filter blockage, whereas both EVS and IVS were prominent for abnormal valve clearance, with greater attention to IVS. CDCFN_V/A therefore captured both general engine impacts and fault-specific features, consistent with its higher recognition accuracy.

#### 4.5.2. LayerCAM Analysis for Dataset II: Mechanical Faults

**Figure 20:**
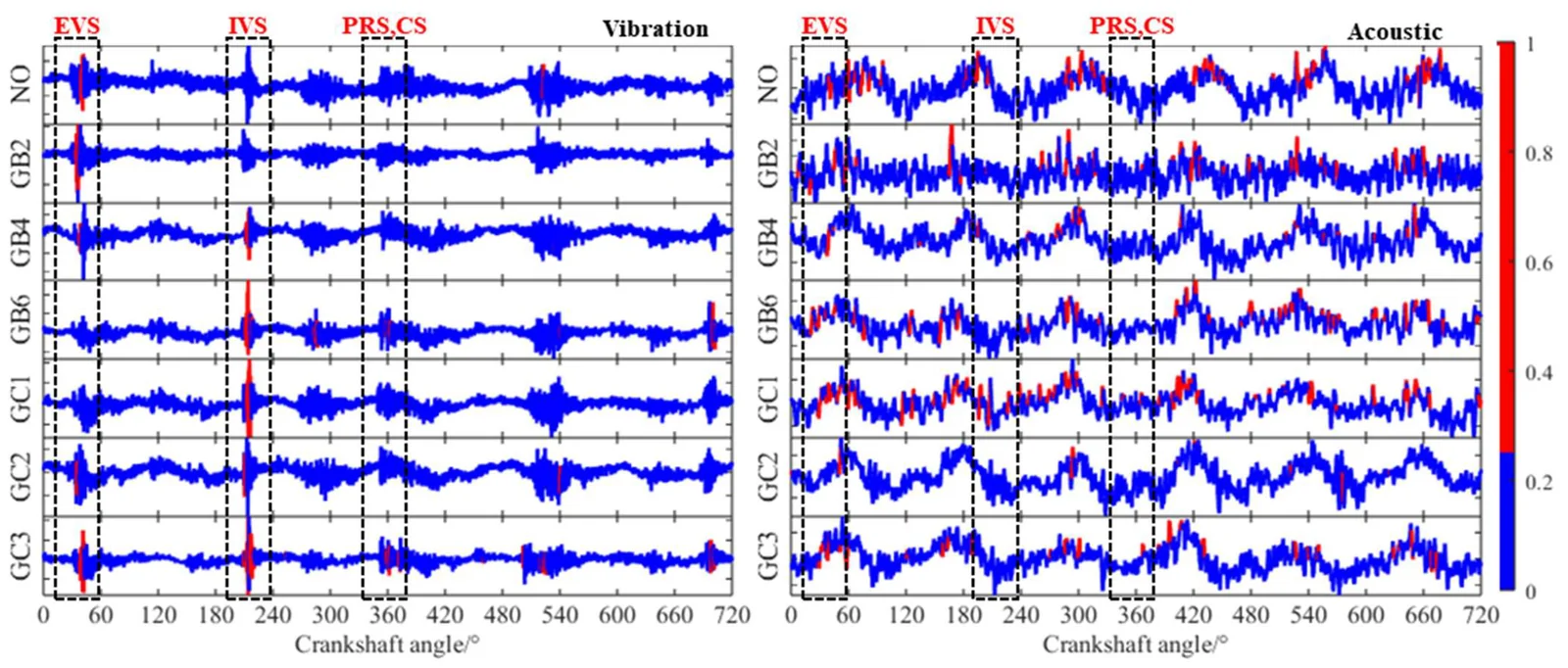
LayerCAM visualisations of CDCFN_A/V for Dataset II.

**Figure 21:**
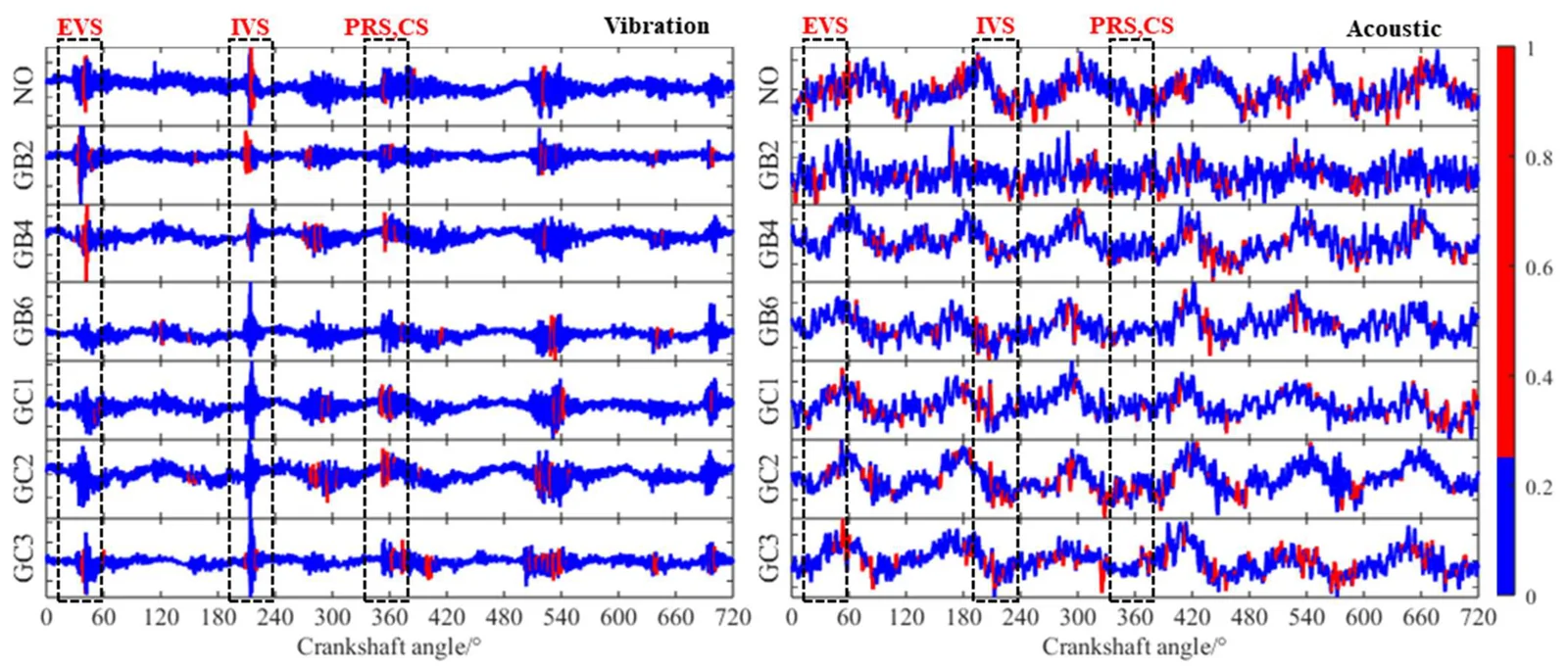
LayerCAM visualisations of CDCFN_V/A for Dataset II.

At 1200 RPM and 50% load, CDCFN_A/V and CDCFN_V/A achieved 96.43% and 97.29% accuracy on Dataset II, respectively. Figure 20 and Figure 21 show their activation maps. Cracks and broken teeth in the timing gear disturb torque transmission and the timing of intake, compression, combustion, and exhaust. The vibration maps contain concentrated activations around the resulting impact components, whereas the acoustic maps contain fewer distinct features.

Figure 20 shows that CDCFN_A/V attended to EVS, IVS, PRS, and CS in the vibration signals. EVS received the greatest attention under normal operation. EVS and IVS were prominent for timing-gear cracks, whereas IVS dominated for broken teeth. CDCFN_A/V therefore captured the principal engine impacts but placed less emphasis on the full set of fault-specific timing features.

Figure 21 shows that CDCFN_V/A attended to EVS, IVS, PRS, and CS. All four components were prominent under normal operation and contributed to both crack and broken-tooth recognition. Relative to the operating-cycle analysis in Figure 6, the model captured multiple timing-related impacts induced by the mechanical faults, consistent with its stronger recognition performance.

## 5. Conclusions

CDCFN was developed for small-sample vibro-acoustic diagnosis of diesel-engine faults across multiple tested operating and matched-noise conditions. Modality-specific convolutional modules extract vibration and acoustic features, MCAM applies separate temporal attention followed by joint channel attention, and CMCD forms reciprocal acoustic-weighted and vibration-weighted decisions through cross-modal attention-score pooling. Experiments on thermal- and mechanical-fault datasets evaluated module ablations, matched-noise performance, comparisons with existing models, and the physical plausibility of the learned features.

Across the four tested loads, CDCFN_V/A achieved the highest mean accuracy on the thermal-fault dataset (98.23 ± 0.68%), whereas CDCFN_A/V achieved the highest mean accuracy on the mechanical-fault dataset (97.99 ± 0.63%). The corresponding weighted F1 scores were 98.22% and 98.05%. The 0.13-percentage-point difference between CDCFN_V/A and MCNNCD_V/A on the thermal-fault dataset was small relative to run-to-run variation and is not interpreted as statistically established superiority.

Under matched Gaussian-noise training and testing at −12 dB, the across-load mean accuracies of CDCFN_A/V and CDCFN_V/A were 94.89 ± 0.34% and 54.61 ± 1.65% on the thermal-fault dataset and 74.71 ± 1.45% and 65.60 ± 1.46% on the mechanical-fault dataset. These results show that low-SNR performance depended strongly on the cross-modal weighting direction.

LayerCAM visualisations indicated that CDCFN attended to intake-valve seating, exhaust-valve seating, piston reversal, and combustion responses. These maps provide qualitative evidence that the learned representation aligns with known events in the engine cycle.

Several limitations constrain the interpretation of these results. For each fault-class–load combination, the training and test windows were non-overlapping but originated from the same continuous acquisition; recording-level independence was therefore not established. A complete independent repeat of the multi-fault diesel-engine campaign would require a new acquisition design rather than a simple repartitioning of the present record. All four loads were represented in both sets, so this study evaluates performance across tested loads rather than transfer to an unseen load. The noise study used matched Gaussian white-noise levels in training and testing and did not examine unequal modality-specific SNRs or clean-to-noisy transfer. The computational experiment reports repeated GPU training and test times for the four ablation architectures on Dataset I, but peak GPU/CPU memory, CPU latency, and external-model runtime were not profiled. No paired inferential test or full single-modality control set was conducted. Finally, CMCD performs directional attention-score pooling rather than explicit probabilistic conditional modelling. Future work will prioritise independent repeat acquisitions, leave-one-load-out and cross-engine validation, clean-to-noisy and asymmetric modality-noise tests, single-modality controls, and cross-platform computational profiling.

## Figures and Tables

**Table 1:** CDCFN model parameters.

Input	Vibration Modal: (1, 5120)	Acoustic Modal: (1, 5120)
Block1	Conv (1, 32, 32), BN, ReLU	Conv (1, 32, 32), BN, ReLU
	Max pooling (2, 2)	Max pooling (2, 2)
	Conv (32, 4, 3), BN, ReLU	Conv (32, 4, 3), BN, ReLU
	MCAM (4 + 4, 4 + 4)	
Block2	Conv (4, 8, 3), BN, ReLU	Conv (4, 8, 3), BN, ReLU
	Max pooling (2, 2)	Max pooling (2, 2)
	Conv (8, 8, 3), BN, ReLU	Conv (8, 8, 3), BN, ReLU
	MCAM (8 + 8, 8 + 8)	
Block3	Conv (8, NumClass, 3), BN, ReLU	Conv (8, NumClass, 3), BN, ReLU
	Max pooling (2, 2)	Max pooling (2, 2)
	Conv (NumClass, NumClass, 3), BN, ReLU	Conv (NumClass, NumClass, 3), BN, ReLU
	MCAM (NumClass + NumClass, NumClass + NumClass)	
Block4	CMCD (NumClass, NumClass, NumClass, NumClass)	
Output	GAP (NumClass)	

Notes: Conv denotes convolution and is specified as input channels, output channels, and kernel size. Unless otherwise stated, convolutional layers use a stride of 1 and padding of 1. Max pooling is specified by pooling size and stride. In MCAM, ‘4 + 4’ denotes concatenation of four vibration-feature channels and four acoustic-feature channels. BN denotes batch normalisation, and NumClass is the number of fault classes.

**Table 2:** Engine operating conditions.

Working Speed (r/min)	Throttle Opening (%)	Torque (N·m)	Power (kW)
1200	0	0	0
	25	566	71
	50	1085	135
	75	1386	174

**Table 3:** Thermal-fault configurations.

Number	Description	Abbreviation
1	No fault in the engine	NO
2	Air intake filter blocking 20%	B20
3	Air intake filter blocking 40%	B40
4	Air intake filter blocking 60%	B60
5	Intake valve clearance decreased by 0.21 mm	D21
6	Intake valve clearance decreased by 0.13 mm	D13
7	Intake valve clearance decreased by 0.05 mm	D05
8	Intake valve clearance increased by 0.05 mm	I05
9	Intake valve clearance increased by 0.12 mm	I12
10	Intake valve clearance increased by 0.19 mm	I19

**Table 4:** Mechanical-fault configurations.

Number	Description	Abbreviation
1	No fault in the engine	NO
2	Timing gear breaking 2 mm	GB2
3	Timing gear breaking 4 mm	GB4
4	Timing gear breaking 6 mm	GB6
5	Timing gear cracking 1 mm	GC1
6	Timing gear cracking 2 mm	GC2
7	Timing gear cracking 3 mm	GC3

**Table 5:** Recognition results for Dataset I.

Method	Index	Accuracy/%	Precision/%	Recall/%	F1 Score/%	Kappa/%
MCNN	0 Load	92.31 ± 3.00	93.70 ± 2.32	92.31 ± 3.00	92.00 ± 3.22	91.46 ± 3.34
	25 Load	95.66 ± 1.78	96.26 ± 1.22	95.66 ± 1.78	95.61 ± 1.80	95.18 ± 1.98
	50 Load	77.98 ± 5.37	83.48 ± 3.81	77.98 ± 5.37	76.45 ± 6.19	75.53 ± 5.97
	75 Load	90.12 ± 2.77	92.31 ± 1.52	90.12 ± 2.77	89.53 ± 3.18	89.02 ± 3.08
	Average	89.02 ± 3.23	91.44 ± 2.22	89.02 ± 3.23	88.40 ± 3.60	87.80 ± 3.59
MCNNCD_A/V	0 Load	98.78 ± 0.65	98.91 ± 0.56	98.78 ± 0.65	98.77 ± 0.66	98.64 ± 0.72
	25 Load	95.36 ± 0.54	95.97 ± 0.52	95.36 ± 0.54	95.26 ± 0.57	94.84 ± 0.60
	50 Load	82.75 ± 0.53	84.64 ± 0.82	82.75 ± 0.53	82.38 ± 0.56	80.83 ± 0.59
	75 Load	98.39 ± 0.40	98.47 ± 0.37	98.39 ± 0.40	98.38 ± 0.40	98.21 ± 0.44
	Average	93.82 ± 0.53	94.50 ± 0.57	93.82 ± 0.53	93.70 ± 0.55	93.13 ± 0.59
MCNNCD_V/A	0 Load	98.71 ± 0.61	98.76 ± 0.58	98.71 ± 0.61	98.70 ± 0.62	98.57 ± 0.68
	25 Load	98.80 ± 0.44	98.87 ± 0.4	98.80 ± 0.44	98.80 ± 0.44	98.67 ± 0.49
	50 Load	97.49 ± 0.51	97.62 ± 0.47	97.49 ± 0.51	97.49 ± 0.50	97.21 ± 0.56
	75 Load	97.40 ± 0.66	97.60 ± 0.57	97.40 ± 0.66	97.39 ± 0.66	97.11 ± 0.73
	Average	98.10 ± 0.56	98.21 ± 0.50	98.10 ± 0.56	98.10 ± 0.56	97.89 ± 0.62
DCFN	0 Load	94.57 ± 1.94	95.36 ± 1.38	94.57 ± 1.94	94.36 ± 2.14	93.97 ± 2.16
	25 Load	92.39 ± 2.40	93.70 ± 1.70	92.39 ± 2.40	92.00 ± 2.74	91.54 ± 2.67
	50 Load	86.55 ± 2.36	88.42 ± 1.43	86.55 ± 2.36	86.11 ± 2.53	85.06 ± 2.62
	75 Load	87.95 ± 4.13	90.94 ± 2.67	87.95 ± 4.13	87.20 ± 4.77	86.61 ± 4.59
	Average	90.37 ± 2.71	92.10 ± 1.80	90.37 ± 2.71	89.92 ± 3.05	89.29 ± 3.01
CDCFN_A/V	0 Load	99.23 ± 0.37	99.27 ± 0.34	99.23 ± 0.37	99.23 ± 0.37	99.14 ± 0.41
	25 Load	96.24 ± 1.20	96.57 ± 1.04	96.24 ± 1.20	96.14 ± 1.27	95.82 ± 1.34
	50 Load	82.69 ± 1.14	84.53 ± 1.00	82.69 ± 1.14	82.41 ± 1.15	80.77 ± 1.27
	75 Load	98.57 ± 0.41	98.64 ± 0.38	98.57 ± 0.41	98.56 ± 0.41	98.41 ± 0.46
	Average	94.18 ± 0.78	94.75 ± 0.69	94.18 ± 0.78	94.09 ± 0.80	93.54 ± 0.87
CDCFN_V/A	0 Load	99.59 ± 0.21	99.6 ± 0.21	99.59 ± 0.21	99.59 ± 0.21	99.54 ± 0.24
	25 Load	98.40 ± 0.77	98.48 ± 0.73	98.40 ± 0.77	98.39 ± 0.78	98.22 ± 0.86
	50 Load	97.57 ± 0.62	97.69 ± 0.59	97.57 ± 0.62	97.57 ± 0.63	97.30 ± 0.69
	75 Load	97.34 ± 1.11	97.50 ± 1.01	97.34 ± 1.11	97.33 ± 1.12	97.04 ± 1.23
	Average	98.23 ± 0.68	98.31 ± 0.63	98.23 ± 0.68	98.22 ± 0.68	98.03 ± 0.76

**Table 6:** Recognition results for Dataset II.

Method	Load	Accuracy/%	Precision/%	Recall/%	F1 Score/%	Kappa/%
MCNN	0	91.53 ± 9.03	94.6 ± 3.85	91.53 ± 9.03	90.45 ± 10.53	90.12 ± 10.54
	25	88.39 ± 10.43	93.24 ± 5.14	88.39 ± 10.43	87.37 ± 11.56	86.45 ± 12.17
	50	93.51 ± 3.11	94.93 ± 2.23	93.51 ± 3.11	93.28 ± 3.34	92.43 ± 3.63
	75	93.71 ± 4.66	95.70 ± 2.78	93.71 ± 4.66	93.27 ± 5.17	92.67 ± 5.43
	Average	91.79 ± 6.81	94.62 ± 3.50	91.79 ± 6.81	91.09 ± 7.65	90.42 ± 7.94
MCNNCD_A/V	0	98.21 ± 2.12	98.54 ± 1.64	98.21 ± 2.12	98.14 ± 2.24	97.92 ± 2.47
	25	98.26 ± 1.61	98.56 ± 1.20	98.26 ± 1.61	98.23 ± 1.66	97.97 ± 1.88
	50	90.49 ± 2.28	92.66 ± 2.05	90.49 ± 2.28	90.68 ± 2.31	88.90 ± 2.66
	75	99.97 ± 0.06	99.97 ± 0.06	99.97 ± 0.06	99.97 ± 0.06	99.97 ± 0.07
	Average	96.73 ± 1.52	97.43 ± 1.24	96.73 ± 1.52	96.76 ± 1.57	96.19 ± 1.77
MCNNCD_V/A	0	97.71 ± 1.33	97.82 ± 1.25	97.71 ± 1.33	97.69 ± 1.36	97.33 ± 1.55
	25	97.70 ± 1.00	97.83 ± 0.93	97.70 ± 1.00	97.66 ± 1.03	97.32 ± 1.17
	50	94.13 ± 0.96	94.55 ± 0.91	94.13 ± 0.96	94.05 ± 1.02	93.15 ± 1.12
	75	98.17 ± 0.85	98.21 ± 0.82	98.17 ± 0.85	98.15 ± 0.86	97.87 ± 0.99
	Average	96.93 ± 1.03	97.1 ± 0.98	96.93 ± 1.03	96.89 ± 1.07	96.42 ± 1.21
DCFN	0	99.19 ± 1.23	99.23 ± 1.16	99.19 ± 1.23	99.18 ± 1.25	99.05 ± 1.44
	25	99.26 ± 0.47	99.28 ± 0.45	99.26 ± 0.47	99.26 ± 0.47	99.13 ± 0.55
	50	95.07 ± 1.25	95.37 ± 1.38	95.07 ± 1.25	95.00 ± 1.28	94.25 ± 1.46
	75	97.74 ± 1.64	98.14 ± 1.17	97.74 ± 1.64	97.72 ± 1.68	97.37 ± 1.91
	Average	97.81 ± 1.15	98.01 ± 1.04	97.81 ± 1.15	97.79 ± 1.17	97.45 ± 1.34
CDCFN_A/V	0	99.91 ± 0.15	99.92 ± 0.14	99.91 ± 0.15	99.91 ± 0.15	99.90 ± 0.17
	25	99.80 ± 0.29	99.81 ± 0.28	99.8 ± 0.29	99.8 ± 0.29	99.77 ± 0.34
	50	92.29 ± 2.03	94.48 ± 1.35	92.29 ± 2.03	92.54 ± 1.94	91.00 ± 2.37
	75	99.96 ± 0.07	99.96 ± 0.06	99.96 ± 0.07	99.96 ± 0.07	99.95 ± 0.08
	Average	97.99 ± 0.63	98.54 ± 0.46	97.99 ± 0.63	98.05 ± 0.61	97.65 ± 0.74
CDCFN_V/A	0	98.36 ± 1.31	98.48 ± 1.14	98.36 ± 1.31	98.32 ± 1.36	98.08 ± 1.52
	25	97.49 ± 1.51	97.64 ± 1.37	97.49 ± 1.51	97.44 ± 1.56	97.07 ± 1.76
	50	94.47 ± 1.59	94.93 ± 1.32	94.47 ± 1.59	94.39 ± 1.64	93.55 ± 1.85
	75	98.99 ± 0.47	99.01 ± 0.46	98.99 ± 0.47	98.98 ± 0.47	98.82 ± 0.55
	Average	97.33 ± 1.22	97.52 ± 1.07	97.33 ± 1.22	97.28 ± 1.26	96.88 ± 1.42

**Table 7:** Computational time of the ablation architectures on Dataset I.

Model	Training Time for 100 Epochs (s)	Test Time for 1000 Samples (s)	Inference Time per Sample (ms)
MCNN	117.34 ± 0.09	0.448 ± 0.004	0.448
MCNNCD	167.32 ± 1.34	0.689 ± 0.005	0.689
DCFN	186.37 ± 18.35	0.871 ± 0.162	0.871
CDCFN	186.69 ± 1.81	0.749 ± 0.018	0.749

## Data Availability

The data presented in this study are available from the corresponding author upon reasonable request.
